# Distribution of alternative untranslated regions within the mRNA of the *CELF1* splicing factor affects its expression

**DOI:** 10.1038/s41598-021-03901-9

**Published:** 2022-01-07

**Authors:** Arkadiusz Kajdasz, Daria Niewiadomska, Michal Sekrecki, Krzysztof Sobczak

**Affiliations:** Department of Gene Expression, Institute of Molecular Biology and Biotechnology, Faculty of Biology, Adam Mickiewicz University Poznan, Uniwersytetu Poznanskiego 6, 61-614 Poznan, Poland

**Keywords:** RNA metabolism, Neuromuscular disease

## Abstract

CUG-binding protein, ELAV-like Family Member 1 (CELF1) plays an important role during the development of different tissues, such as striated muscle and brain tissue. CELF1 is an RNA-binding protein that regulates RNA metabolism processes, e.g., alternative splicing, and antagonizes other RNA-binding proteins, such as *Muscleblind*-like proteins (MBNLs). Abnormal activity of both classes of proteins plays a crucial role in the pathogenesis of myotonic dystrophy type 1 (DM1), the most common form of muscular dystrophy in adults. In this work, we show that alternative splicing of exons forming both the 5′ and 3′ untranslated regions (UTRs) of *CELF1* mRNA is efficiently regulated during development and tissue differentiation and is disrupted in skeletal muscles in the context of DM1. Alternative splicing of the *CELF1* 5′UTR leads to translation of two potential protein isoforms that differ in the lengths of their N-terminal domains. We also show that the MBNL and CELF proteins regulate the distribution of mRNA splicing isoforms with different 5′UTRs and 3′UTRs and affect the *CELF1* expression by changing its sensitivity to specific microRNAs or RNA-binding proteins. Together, our findings show the existence of different mechanisms of regulation of *CELF1* expression through the distribution of various 5′ and 3′ UTR isoforms within *CELF1* mRNA.

## Introduction

The strict control of every step of gene expression at the RNA level, such as transcription, mRNA maturation, transport, stability and translation, plays a crucial role during development, tissue differentiation and pathological processes. Many RNA-binding proteins (RBPs) regulate one or multiple of these steps through interactions with specific groups of transcripts. One such protein is CUG binding protein, ELAV-like Family member 1 (CELF1). CELF1 was isolated from HeLa cell extract as a protein that binds to CUG triplet repeats within RNA^1^. It belongs to the family of CELF proteins, which consists of six members^2,3^. CELFs are ubiquitously expressed and are important during cell cycle progression and tissue development. CELFs bind to many RNA targets and control RNA processing at different stages. Each CELF features three highly conserved RNA recognition motif (RRM) domains^3^. Two of them are located on the N-terminus of the protein, and the third is located on the C-terminus. These domains are separated by divergent domains that are unique to each CELF member. CELF1 and CELF2 are members of one subfamily, while CELF3-6 belong to a second subfamily. Nuclear localization signals are located on the C-termini of all CELFs^4^. CELF1-2 are common in most embryonic tissues, whereas CELF3-6 are present in adult tissues, mostly in the nervous system^3,5,6^.

CELF1 may regulate RNA metabolism by binding to GU-rich elements (GREs) in both the nucleus and cytoplasm^7,8^ in three different ways: (1) through alternative splicing regulation by binding to pre-mRNAs^8^; (2) through translation activation or inhibition by binding to the 5′ untranslated regions (5′UTRs) of many mRNAs (e.g., p21, C/EBPB, and MEF2A)^9,10^; and (3) through RNA decay by binding to 3′UTR sequences^8,11,12^ or mature microRNAs (miRNAs)^13^ and recruiting appropriate nucleases that deadenylate targeted transcripts. CELF1 is a critical splicing factor during muscle development. Its level is high in embryos and is significantly reduced in postnatal development, especially during the first days after birth, often without changes in *CELF1* mRNA levels^14^. The mechanism of these changes is still unknown.

CELF1 protein plays an important role in the pathogenesis of myotonic dystrophy type 1 (DM1)^1,15^. DM1 is an autosomal dominant multisystem disease, although the first symptoms usually involve skeletal and heart muscles^16^. DM1 is caused by an expansion of trinucleotide CTG repeats located in the 3′UTR of the Dystrophia Myotonica Protein Kinase gene (*DMPK*)^17^. Mutant mRNA containing hundreds or thousands CUG repeats is transcribed and undergoes proper maturation^18^. Expanded CUG repeat tract forms a stable RNA hairpin structure and sequesters *Muscleblind*-Like proteins (MBNLs), which are regulators of alternative splicing and polyadenylation^19,20^. These complexes remain within nuclei. In DM1, CELF1 undergoes hyperphosphorylation by Protein Kinase C (PKC), leading to higher stability and relocation mainly to cell nuclei^21^. MBNL lack-of-function and CELF1 gain-of-function are the main causes of global abnormalities in alternative splicing and alternative polyadenylation^22^ in different tissues of DM1 patients. In some cases, these abnormalities are intensified because MBNLs and CELF1 mostly play opposing roles in splicing regulation of individual alternative exons^8^. In the muscles of adults with DM1, the splicing patterns of many mRNAs are similar to those observed in normal muscles during the prenatal-postnatal transition. Changes in the alternative splicing of many genes, e.g., *CLCN1*, *BIN1*, *INSR* and *PKM*, cause characteristic DM1 symptoms such as myotonia, progressive muscle wasting, insulin resistance and cardiac arrhythmia, respectively^23–27^.

In this study, we showed that the sequences of both the 5′UTR and 3′UTR of *CELF1* are highly variable and are regulated by different posttranscriptional mechanisms during the development of many tissues and in the context of DM1. Alternative splicing of the *CELF1* 5′UTR leads to translation of two mostly nuclear CELF1 protein isoforms with unknown differences in their functions. Moreover, mRNA splicing isoforms with distinct 3′UTRs are under the pressure of specific miRNAs and RBPs. MBNL proteins and CELF itself act as regulators of CELF1 quantity by changing UTR sequences in *CELF1* mRNA isoforms. These findings suggest that a significantly different steady state level of CELF1 protein could be observed in the cells regardless of the level of its mRNA.

## Results

### The 5′ and 3′ UTRs of *CELF1* are alternatively spliced in different tissues and muscles of DM1

**Figure 1:**
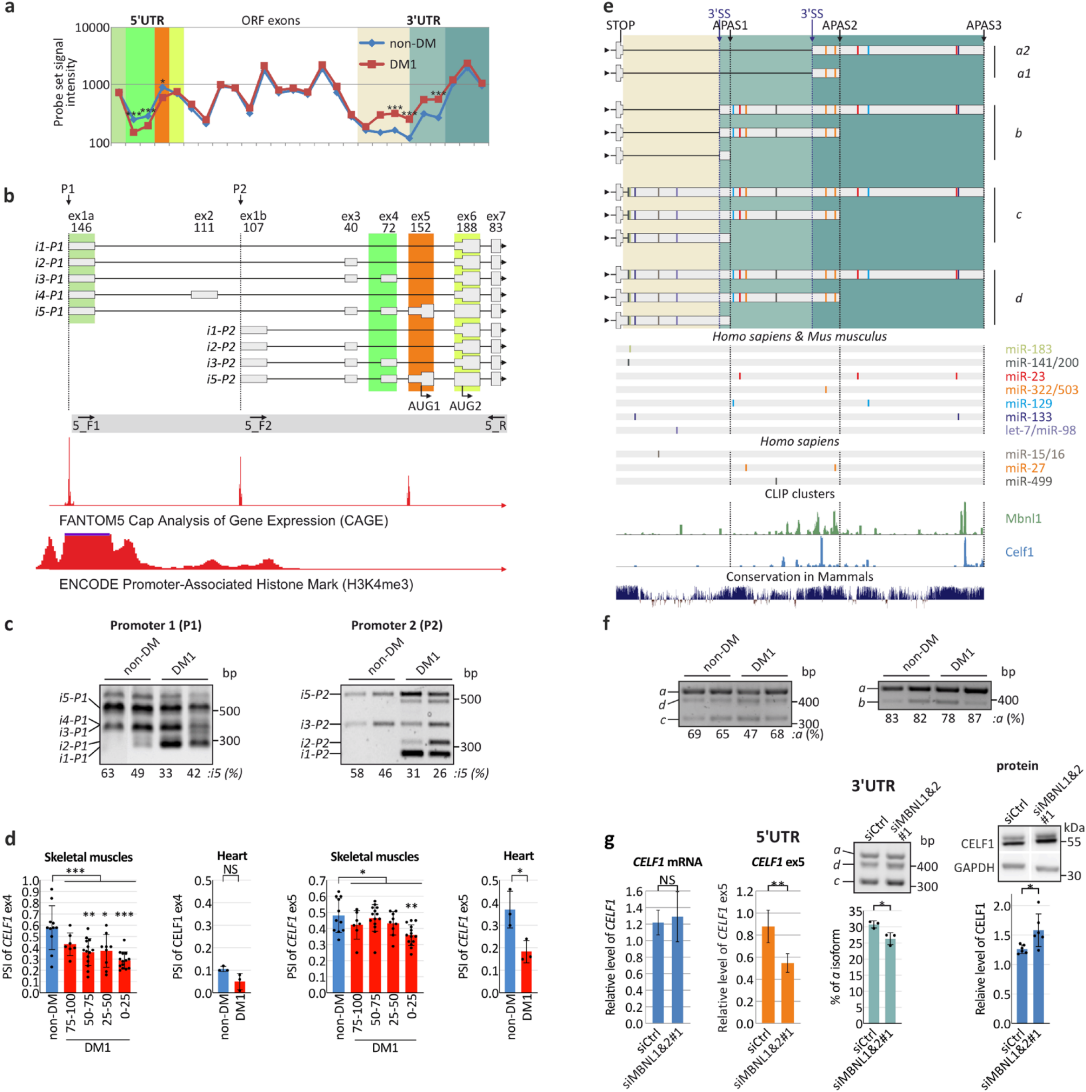
Changes in the composition of *CELF1* 5′UTR and 3′UTR in DM1. (**a**) Distribution of *CELF1* exons in DM1 based on the results of Affymetrix All-Exon Array experiments^28^. The chart shows the hybridization signals of probe sets representing different *CELF1* exons in non-DM (blue line and diamonds) and DM1 (red line and squares) skeletal muscle samples. Significant changes were observed for exons forming the 5′ and 3′UTRs but not for those forming the open reading frame (ORF; white background). The probe sets representing these regions are marked by colorful backgrounds and correspond to the exons represented by gray boxes in the cartoons (**b** and Fig. 2a). (**b**) The scheme of putative *CELF1* 5′UTR isoforms. Based on analysis of the 5′ ends of transcripts (FANTOM5 CAGE) and ENCODE Promoter-Associated Histone Mark (H3K4Me3)^58^ we distinguished two putative *CELF1* transcription start sites (TSSs) in skeletal muscles (P1 and P2). *CELF1* 5′UTR may vary in length and exon composition due to alternative TSS usage and alternative splicing. Sequencing of RT-PCR products revealed five *CELF1* 5′UTR isoforms (*i1*–*i5*) transcribed from the first promoter (P1) and four isoforms (*i1, i2, i3,* and *i5*) transcribed from the second promoter (P2). Two translation start codons are marked (AUG1 and AUG2), and the values under the exon numbers (ex) indicate their lengths in nucleotides. Arrows show primer localizations. Lower boxes, UTR exons; upper boxes, ORF exons; lines, introns. (**c**) RT-PCR analysis of alternative splicing of *CELF1* 5′UTR in skeletal muscle samples of non-DM and DM1 patients (left panel: primers 5_F1 and 5_R; right panel: primers 5_F2 and 5_R). The splicing in this region of mRNAs transcribed from P1 (left panel) or P2 (right panel) is disrupted. In both cases, the shortest isoform (*i1*) lacking alternative cassette exons predominates (P = 0.02 for P1 and P = 0.003 for P2, *t* test), while isoform 5 (*i5*) containing ex5 (AUG1) is less prevalent. Framed gels are presented. The unprocessed, full-sized images cannot be provided in Supplementary Information due to storage server damage. (**d**) Alternative splicing of *CELF1* ex4 and ex5 in DM1 skeletal muscles (*tibialis anterior*) divided into the subgroups according to a normalized ankle dorsiflexion strength expressed in % (non-DM, *n* = 11; DM1 75–100, *n* = 7; DM1 50–75, *n* = 14; DM1 25–50, *n* = 9; DM1 0–25, *n* = 14)^31^ and heart (non-DM, *n* = 3; DM1, *n* = 3)^31,32^ based on RNA-Seq data. PSI, percent spliced in index. (**e**) Alternative splicing (3′SS, 3′ splice site) and alternative polyadenylation sites (APASs) lead to the generation of at least eleven potential *CELF1* 3′UTR isoforms (*a*, *b*, *c*, and *d*). Below the isoform structures, the miRNA target sites predicted for *Homo sapiens* and *Mus musculus* (based on TargetScan^59^, TarBase^60^, miRanda^61^, PicTar^62^, miRTarBase^63^ and the papers by Kalsotra et al*.*^38^ and Dong et al*.*^41^, the Mbnl1 and Celf1 binding sites predicted based on crosslinking immunoprecipitation (CLIP)-Seq results^8,42,64^ and the sequence conservation are shown. (**f**) RT-PCR analysis of *CELF1* 3′UTR isoforms distribution in DM1 skeletal muscles (left panel: primers 3_F1, 3_Ra and 3_Rcd; right panel: primers 3_F1, 3_Ra and 3_Rb). Framed gels are presented. The unprocessed, full-sized images cannot be provided in Supplementary Information due to storage server damage. (**g**) The expression of *CELF1* is under the control of MBNLs. Knockdown of *MBNL1* and *MBNL2* has no effect on *CELF1* mRNA level (normalized to *GAPDH*); although, decreases the inclusion of ex5 (real-time RT-PCR; primers 5_F3 and 5_R1, normalized to all *CELF1* transcripts) (siCtrl, *n* = 5; siMBNL1&2#1, *n* = 5). The distributions of *CELF1* 3′UTR isoforms are sensitive to MBNL protein level (primers 3_F1, 3_Ra and 3_Rcd) (*n* = 3); cropped gels are presented. CELF1 protein (normalized to GAPDH) is upregulated in MBNLs deficiency conditions (siCtrl, *n* = 6; siMBNL1&2#1, *n* = 6); cropped blots are presented. The efficiency of *MBNL1* and *MBNL2* knockdown is presented in Supplementary Fig. S2c. Statistical significance was calculated with unpaired, two-tailed *t* test (*NS* non-significant; *P < 0.05; **P < 0.01 and ***P < 0.001).

In a recent study, we investigated the global changes in the transcriptomes of DM patients. In skeletal muscle biopsies obtained from 16 non-DM individuals and 15 DM patients, we identified hundreds of alternative exons showing significant differences in distribution within mRNAs due to abnormalities in alternative splicing or alternative polyadenylation^22,28^. *CELF1* mRNA was one of the affected transcripts. The results of whole-transcriptome analysis suggested that both the 5′ and 3′ UTRs of this mRNA undergo abnormal expression in skeletal muscles in the context of DM1. The inclusion rate of exons 4 and 5 (further referred to as ex4 and ex5), which form the 5′UTR, was significantly attenuated in DM1, whereas the inclusion of the inner part of the very long 3′UTR was increased (Fig. 1a). The hybridization signals from probes representing internal exons coding for CELF1 protein were unchanged.

Using a splicing-sensitive RT-PCR assay (primer positions are indicated in Fig. 1b, Supplementary Fig. S1), we confirmed alterations in splicing within the 5′UTR of *CELF1* mRNA. Upon sequencing the PCR products, we identified at least five distinct 5′UTR splicing isoforms (Fig. 1b). Isoform 1 (*i1-P1*) contained spliced ex1 and 6, while isoform 2 (*i2-P1*) was 40 nucleotides longer and contained ex1, 3 and 6. Isoforms 3 (*i3-P1*) and 4 (*i4-P1*) differed in length by only one nucleotide and were composed of ex1, 3, 4 and 6 and ex1, 2 and 6, respectively. Isoforms 1 through 4 differed only in the sequences of their 5′UTRs, whereas isoform 5 had a unique in-frame translation start codon, resulting in the production of a CELF1 protein isoform 27 amino acids longer than the others. Isoform *i1-P1* was expressed at the higher level in DM1 tissues than in non-DM tissues (Fig. 1c, left panel).

Based on ENCODE Promoter-Associated Histone Mark (H3K4me3)^29^ and FANTOM5 Cap Analysis of Gene Expression (CAGE) data^30^, we identified an alternative *CELF1* transcription start site (promoter 2; P2) located within intron 1 (Fig. 1b). All four potential *CELF1* 5′UTR splicing isoforms (*i1-P2*, *i2-P2*, *i3-P2* and *i5-P2*) originating from pre-mRNA transcribed from P2 were confirmed by sequencing of RT-PCR products (Fig. 1c, right panel). These isoforms had exon compositions similar to those of isoforms transcribed from promoter 1 (P1; Fig. 1b, upper panel). Alternative splicing of exons forming *CELF1* 5′UTRs from P2 was also disrupted in DM1; in mRNAs from both promoters, ex5 (containing an alternative AUG codon) was mostly excluded, and the expression of shorter isoforms (mainly isoforms *i1-P1, i1-P2* and *i2-P2*) was increased significantly (Fig. 1c).

The above-mentioned observations were supported by the previously published RNA-Seq data obtained for biopsies of several DM1 and non-DM individuals^31,32^ which suggest that splicing of *CELF1* ex4 and ex5, containing alternative AUG codon, is significantly changed in DM1 skeletal muscles (*tibialis anterior*) and heart (Fig. 1d). DM1 skeletal muscle samples were divided into subgroups according to a normalized ankle dorsiflexion strength^31^. Interestingly, *CELF1* ex4 and ex5 are excluded from the mRNA in patients with more severe DM1 symptoms (Fig. 1d). *CELF1* ex5 is significantly excluded in DM1 heart in comparison to non-DM tissue, whereas *CELF1* ex4 is unchanged (Fig. 1d).

In DM1, the 3′UTR of *CELF1* also varied in length. We showed that the sequence of a last exon contained at least three alternative 3′ splice sites that could give rise to four potential splicing isoform groups, namely, *a*, *b*, *c* and *d* (Fig. 1e). In an RT-PCR assay performed with a set of three primers, we did not observe statistically significant changes in the distribution of these isoforms in DM1 tissue, perhaps due to the low number of tested samples and low contribution of some isoforms in skeletal muscles of both patient and healthy individuals (Fig. 1f); however, all predicted 3′UTRs were identified. Taking into consideration the hybridization signal from microarrays for probes representing different 3′UTR isoforms we can conclude that isoform *a* predominates significantly in both DM1 and non-DM samples. Moreover, data from the RefSeq database suggested that two proximal alternative polyadenylation sites (APASs) could increase the number of potential *CELF1* 3′UTR length variants (Fig. 1e).

As functional insufficiency of MBNL is a major cause of alternative splicing abnormalities in DM1 we then investigated whether MBNLs are responsible for *CELF1* 5′UTR and 3′UTR variability. Overexpression of MBNL1 in HeLa cells significantly increased the inclusion of *CELF1* ex5 (Supplementary Fig. S2a, left panel), while knockdown of MBNLs had the opposite effect with no change in the total *CELF1* mRNA level (Fig. 1g and Supplementary Fig. S2a and d). Additionally, we analyzed publicly available RNA-Seq data with MBNLs depletion to show differences in expression of *CELF1* ex5. This exon is significantly excluded from the mature *CELF1* mRNA in six of eight RNA-Seq data sets (Supplementary Table S1). Moreover, silencing and overexpression of *MBNLs* induced downregulation and upregulation of 3′UTR isoforms *a*, respectively (Fig. 1g and Supplementary Fig. S2b and d). We than asked whether the level of the MBNL pool may affect changes in the level of CELF1 protein. It appeared that the knockdown of MBNLs in HeLa cells caused significant upregulation of CELF1 (Fig. 1g, right panel and Supplementary Fig. S2d, right panel). These data suggest that MBNLs regulate the alternative splicing of the *CELF1* 5′UTR and 3′UTR and have an effect on the steady state level of CELF1 protein.

**Figure 2:**
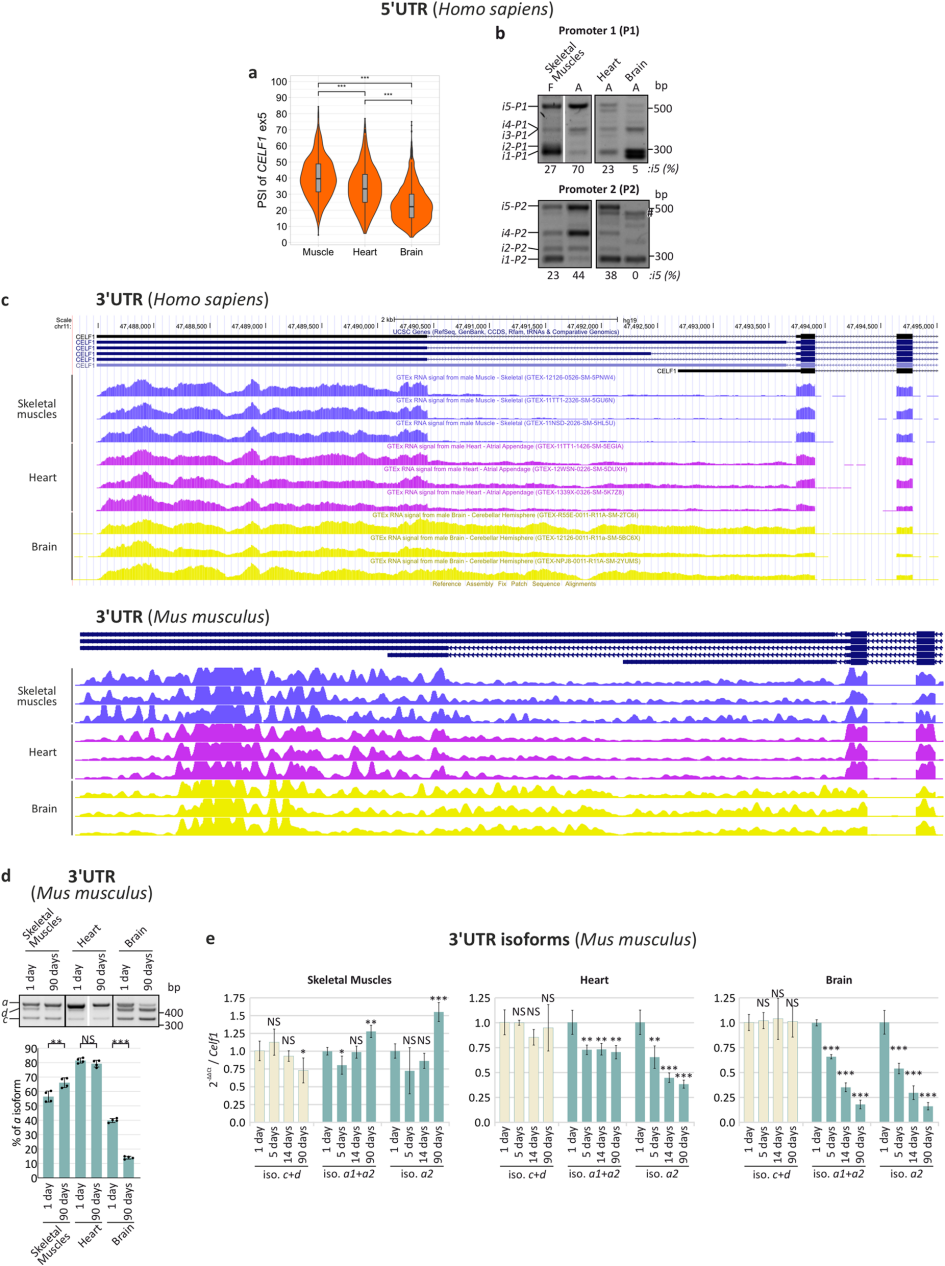
Changes in the composition of *CELF1* 5′UTR and 3′UTR in different human and mouse tissues. (**a**) Alternative splicing of *CELF1* ex5 in human skeletal muscles (*n* = 803), heart (*n* = 861) and brain (*n* = 2642) based on GTEx project RNA-Seq data. PSI, percent spliced in index, calculated according to the following equation, [exon in counts/(exon in counts + exon out counts)] × 100. (**b**) Splicing profiles of *CELF1* 5′UTR during skeletal muscle development (F, fetal; A, adult) and in adult heart and brain tissue (A, adult) (upper panel: primers 5_F1 and 5_R; lower panel: primers 5_F2 and 5_R). The expression of *i5-P1* (upper panel) and *i5-P2* (lower panel) is the highest in adult skeletal muscles and is very low (*i5-P1*) or undetectable (*i5-P2*) in the adult brain. An unspecific RT-PCR product is marked with #; cropped gels are presented. The unprocessed, full-sized images cannot be provided in Supplementary Information due to storage server damage. (**c**) Upper panel, *CELF1* 3′UTR isoforms levels in human tissues. The screenshot from the UCSC genome browser showing GTEx project RNA-Seq data. Lower panel, *CELF1* 3′UTR isoforms levels in mouse tissues. The screenshot from the DM-Seq database^31^. (**d**) RT-PCR analysis of *Celf1* 3′UTR isoforms in mouse skeletal muscles (*n* = 4), heart (*n* = 4) and brain (*n* = 4) during the development in 1 and 90 days after birth (primers 3_F1, 3_Ra and 3_Rcd). Cropped gels are presented. (**e**) Real-time RT-PCR analysis of *Celf1* 3′UTR isoforms in mouse skeletal muscles (*n* = 4), heart (*n* = 4) and brain (*n* = 4) during the development in 1, 5, 14 and 90 days after birth. Isoforms *c* and *d* (primers 3_F1 and 3_Rcd) are downregulated in skeletal muscles, unchanged in heart and brain. Both isoforms *a* are upregulated in skeletal muscles (left panel) and downregulated during heart and brain development (middle and right panel, respectively) (primers 3_F1 and 3_Ra or 3_F1 and 3_R2a). All RT-qPCRs normalized to all *Celf1* transcripts. Statistical significance was calculated with unpaired, two-tailed *t* test (*NS* non-significant; *P < 0.05; **P < 0.01 and ***P < 0.001).

Previously, it has been shown that the expression of *CELF1* occurs predominantly in the brain rather than in heart and skeletal muscles and that the levels of CELF1 protein are significantly decreased during muscle development^3,14,33^. We decided to determine whether posttranscriptional changes in the sequence of the 5′UTR or 3′UTR of *CELF1* occur during the development of mouse and human skeletal muscles, heart and brain. It appeared that alternative splicing of the *CELF1* 5′UTR ex5 significantly differs in these tissues (Fig. 2a,b). The inclusion of *CELF1* ex5, containing alternative AUG1 codon, is the highest in skeletal muscles, lower in heart and the lowest in the brain. The splicing profiles for the mRNAs transcribed from the two promoters in the same samples were slightly different. The longest isoforms, *i5-P1* and *i5-P2*, predominated in skeletal muscles; their levels were much lower in the heart and brain (Fig. 2b). Therefore, it can be concluded that the 5′UTR isoform with exon 5 containing alternative AUG codon is favored in adult skeletal muscles and is reduced in fetal muscles and in DM1.

The distribution of *CELF1* 3′UTR isoforms also varied in different tissues (Fig. 2c,d). During mouse skeletal muscle development, the contribution of isoform *a* constantly increases but isoforms *c* and *d* are downregulated (Fig. 2d,e). During mouse heart and brain development, the percentage of isoform *a* is unchanged or significantly lowered, respectively. A similar pattern of the *CELF1* 3′UTR isoforms is observed in human tissues (Fig. 2c, upper panel).

We also investigated differences in the selection of three alternative polyadenylation sites (APAS1, APAS2 and APAS3) during tissue development. The percentage of transcripts terminated at APAS2 was decreased in mouse and human skeletal muscles during development, resulting in the formation of the longer *CELF1* 3′UTR isoform *a2* (Fig. 2e, left panel)*.* On the contrary, in mouse heart and brain, the *Celf1* 3′UTR isoform *a2* was significantly decreased during the development (Fig. 2e, middle and right panels).

Based on obtained results, we hypothesized that due to sequence alterations, alternative 5′ and 3′UTRs of *CELF1* may affect both the quantity and quality of CELF1 protein in different tissues and in DM1.

### Alternative splicing of the 5′UTR of *CELF1* has no significant effect on the level and activity of CELF1

**Figure 3:**
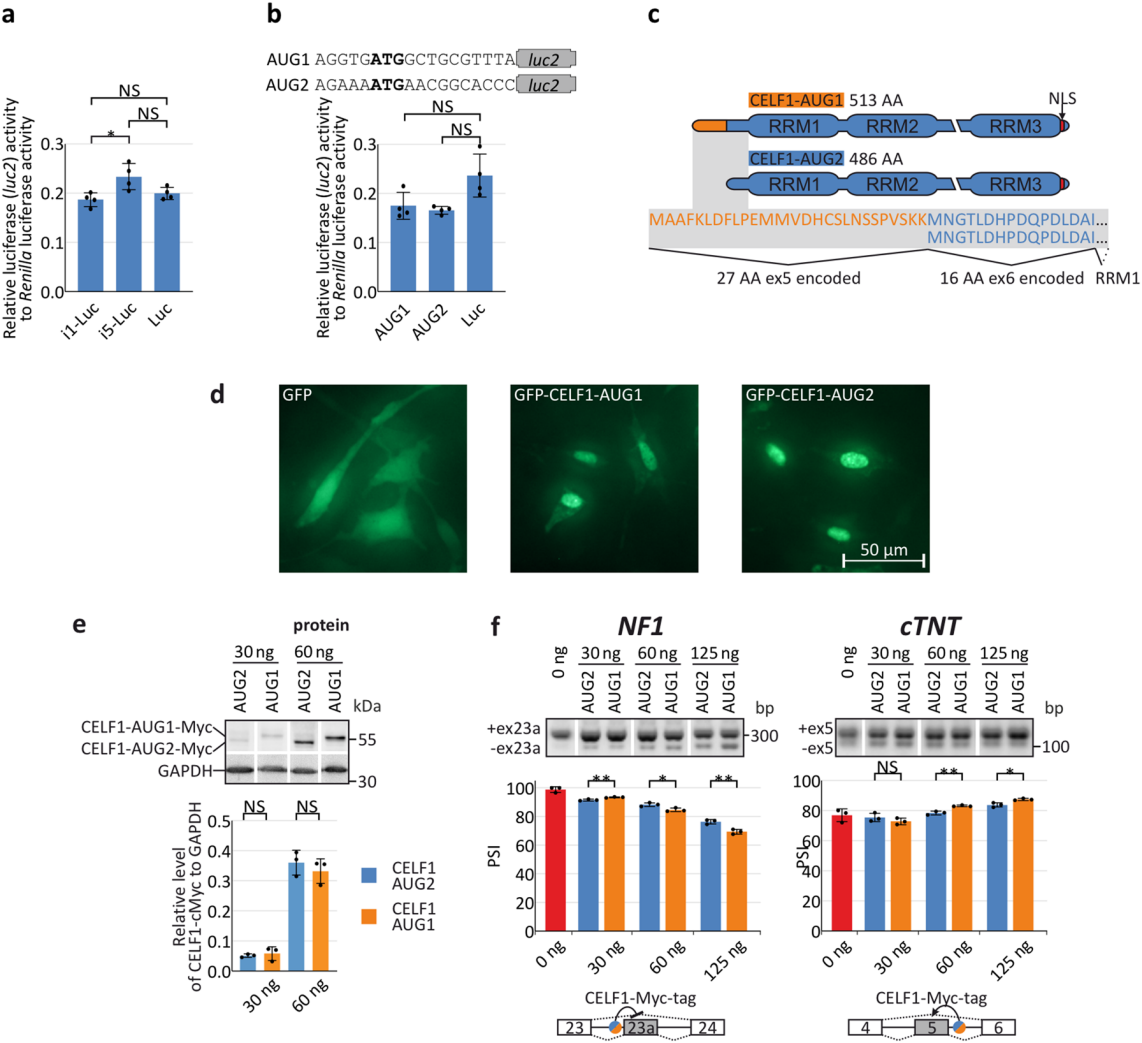
Impact of *CELF1* 5′UTR isoforms on the quantity and activity of CELF1 protein. (**a**) Relative activity of luciferase 2 (*luc2*) translated from plasmids containing *i1-P1* or *i5-P1 CELF1* 5′UTR isoforms. (**b**) Effect of AUG codon context on CELF1 translation activity. Short sequences containing two AUG codons (AUG1 and AUG2) were cloned upstream of the *luc2* ORF (upper panel). Both sequences had similar translation activity (lower panel). (**c**) Two CELF1 protein isoforms differing in length in the N-terminal domain. The CELF1-AUG1 isoform is 27 AA longer than the CELF1-AUG2 isoform; their total lengths are 513 AA and 486 AA, respectively. The nuclear localization signal (NLS) is located on the C-termini of both isoforms. (**d**) The localization of GFP-CELF1-AUG1 and GFP-CELF1-AUG2 in HepG2 cells is the same. (**e**) The transfection with the same amount of plasmid encoding CELF1-AUG1 and CELF1-AUG2 results in the same quantity of translated proteins. Quantification of the level of CELF1-Myc-tag is normalized to GAPDH (COS-7 cells, *n* = 3). Cropped blots are presented. (**f**) Splicing activity of CELF1 isoforms. Different amounts of CELF1-AUG1 or CELF1-AUG2 expression construct were cotransfected with *NF1* and *cTNT* splicing minigenes containing CELF-dependent alternative ex23a and ex5, respectively. The results of RT-PCR splicing assays showed that CELF1-AUG2 (the shorter isoform) had lower splicing activity than CELF1-AUG1 (COS-7 cells, *n* = 3 biological replicates). The lower panels show the CELF1 binding site in intronic region of regulated minigenes. Cropped gels are presented. Statistical significance was calculated with unpaired, two-tailed *t* test (*NS* non-significant; *P < 0.05; **P < 0.01 and ***P < 0.001).

Alternative splicing of *CELF1* 5′UTRs may affect protein production and selection of alternative translation start sites. To explain the functions of different *CELF1* 5′UTR isoforms, we formulated four hypotheses: (1) 5′UTRs affect protein production; (2) two CELF1 protein isoforms have different subcellular localization; (3) they are produced at different levels or (4) have different splicing activity. To investigate the first possibility, we designed two luciferase-expressing constructs containing the most common *CELF1* 5′UTRs differing in alternative exon compositions. The expression of shorter *i1-P1* isoform results in slightly lower protein production than the longer *i5-P1* isoform (Fig. 3a). As CELF1 protein can be translated from two start codons, AUG1 and AUG2 (Fig. 1b), we also investigated whether the activity of the two codons differed. We cloned both AUG codons, within contexts of natural flanking sequences, into the luciferase open reading frame. It appeared that both start codons had similar effects on protein production of the reporter (Fig. 3b). We also checked the effect of MBNL1 and CELF1 overexpression on tested reporter constructs as the level of these proteins are involved in muscle development and DM pathogenesis. They did not affect CELF1 protein production in context of different *CELF1* 5′UTR isoforms (Supplementary Fig. S3a). All these results suggest that the sequence of *CELF1* 5′UTR isoforms and the context of AUG codon have just marginal impact on CELF1 level.

The translation initiation codon AUG2 is present in the constitutive *CELF1* ex6. Inclusion of the alternative ex5 introduces an additional initiation codon, AUG1. Therefore, inclusion of this alternative exon led to synthesis of longer CELF1 protein isoform with 27 extra amino acids at its N-terminus (Fig. 3c). Based on the above observation, we hypothesized that two CELF1 isoforms (AUG1 and AUG2), differing in the presence of an additional 27 AA sequence at N-terminus, have different subcellular localization or are produced on the different level and/or varied in the activity. To test these hypotheses, we generated constructs for overexpression of the two CELF1 variants. Both protein variants, CELF1-AUG1 and CELF1-AUG2, fused with GFP were localized mostly in cell nuclei (Fig. 3d). Then, we transfected COS-7 cells with different amounts (30 and 60 ng) of CELF1 constructs fused with small Myc-tag at C-termini to avoid the potential impact of GFP on their stability (Fig. 3e and Supplementary Fig. S3b). In cells transfected with the same amount of both constructs, the steady state level of CELF1-AUG1 and CELF1-AUG2 was the same. To further check the impact of two CELF1 isoforms on their function, we cotransfected COS-7 cells with different amounts (30–125 ng) of CELF1 expression constructs with *NF1* and *cTNT* splicing minigenes, containing CELF1-dependent alternative exon (Fig. 3f). Previously it was shown that the CELF1 inhibits the inclusion of *NF1* ex23a and promotes the inclusion of *cTNT* ex5 by binding to the upstream or downstream intronic regions of the alternative exons, respectively^34,35^. RT-PCR-based splicing assays showed small but statistically significant differences in splicing regulation by the CELF1 isoforms for the tested minigenes (Fig. 3f). However, observed differences are relatively small and could be caused by other experimental factors, therefore, their biological meaning is inconclusive.

We showed that alternative splicing of the *CELF1* 5′UTR causes the translation of two CELF1 protein isoforms differing in length by 27 amino acids at the N-terminus. However, different *CELF1* 5′UTRs have similar translation activity and both CELF1 proteins show no significant differences in their steady state level and activity. Therefore, the function of two CELF1 protein isoforms remains unknown.

### MiRNAs and CELFs regulate *CELF1* 3′UTR isoform distribution

**Figure 4:**
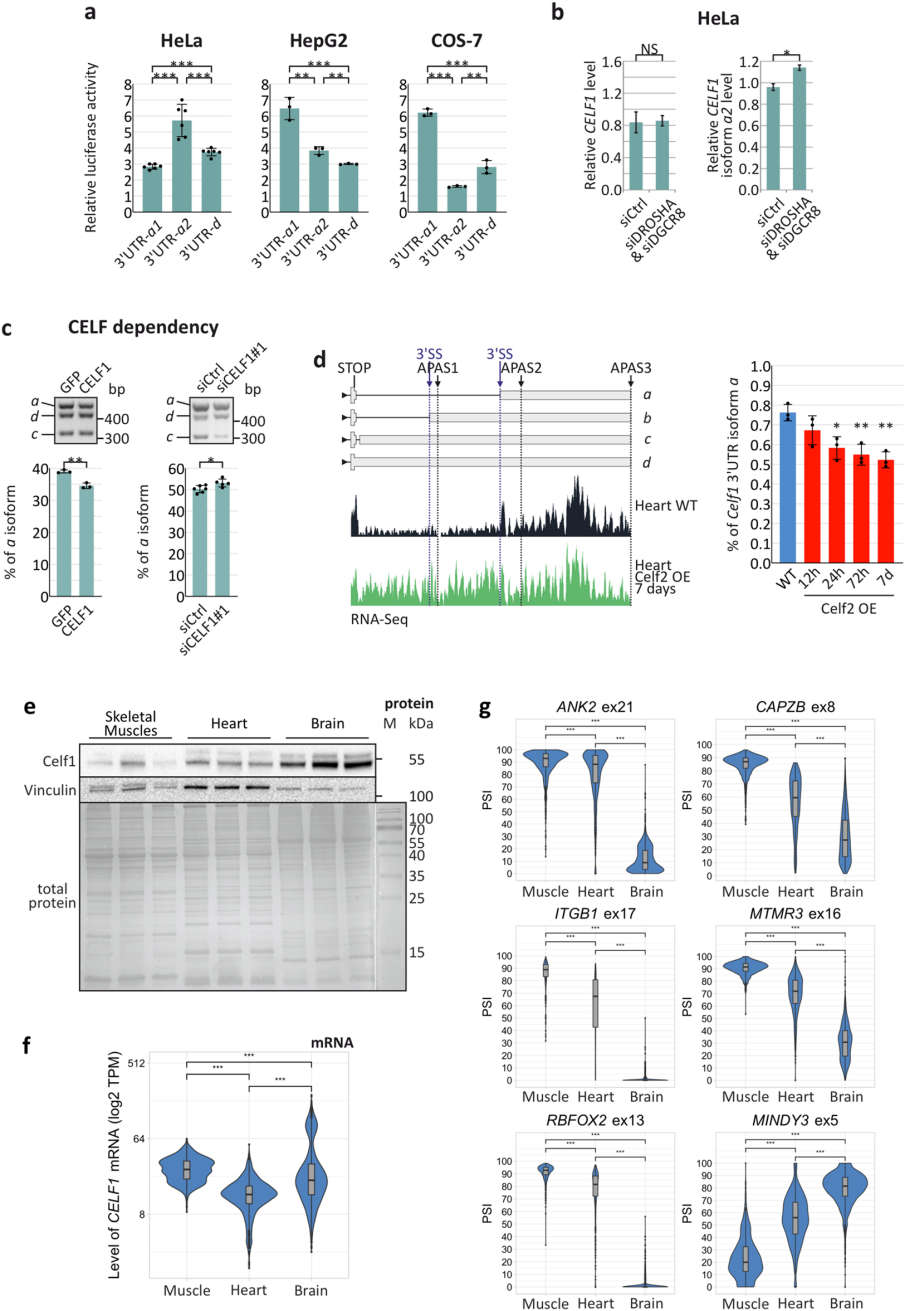
*CELF1* 3′UTR isoform distribution depends on RBPs and miRNAs and impact of *CELF1* 5′UTR and 3′UTR on its expression. (**a**) Three *CELF1* 3′UTR isoforms (*a1*, *a2* and *d*) were cloned downstream of the *luc2* ORF in the pmiR-GLO vector. HeLa (*n* = 6), HepG2 (*n* = 3) and COS-7 (*n* = 3) cells were transfected with these constructs. The results of luciferase activity assays show that different 3′UTR isoforms have different impacts on protein production depending on the sequence of the isoform and the genetic background of the tested cell lines. (**b**) Levels of 3′UTR isoforms of *CELF1* under miRNA deficiency conditions. The total level of *CELF1* mRNA, normalized to *GAPDH*, was unchanged in HeLa cells with miRNA deficiency (cells with knockdown of the microprocessor components *DROSHA* and *DGCR8*); however, based on real-time RT-PCR results the contribution of isoform *a2* (normalized to all *CELF1* transcripts) was higher on the background of miRNA insufficiency (primers 3_F1 and 3_Ra). (**c**) The distributions of *CELF1* 3′UTR isoforms are sensitive to CELF activity (primers 3_F1, 3_Ra and 3_Rcd) (GFP and CELF1, *n* = 3; siCtrl, *n* = 6; siCELF1#1, *n* = 5). Cropped gels are presented. (**d**) RNA-Seq data for the *Celf1* 3′UTR obtained from the hearts of wild-type mice (WT) and mice overexpressing *Celf2* (OE mice)^8,31^. Left panel, scheme represents reads localization in the *Celf1* 3′UTR. Right panel, percentage of *Celf1* 3′UTR isoform *a* in mouse heart 12 h, 24 h, 72 h and 7 days after induction of *Celf2* overexpression. (**e**) Level of Celf1 protein in different murine tissues. 10 µg of protein were loaded per lane. Vinculin was used as a loading control for samples from the same tissue. The total protein is a loading control for samples from different tissues. *M* molecular weight marker. Framed blots and gel are presented. (**f**) *CELF1* mRNA level in skeletal muscles (*n* = 803), heart (*n* = 861) and brain (*n* = 2642) based on GTEx project RNA-Seq data. TPM, transcripts per million. (**g**) Profiles of alternative splicing of CELF1-dependent *ANK2* ex21, *CAPZB* ex8, *ITGB1* ex17, *MTMR3* ex16, *RBFOX2* ex13 (exons negatively regulated by CELF1) and *MINDY3* ex5 (exon positively regulated by CELF1) in different tissues (skeletal muscles, *n* = 803; heart, *n* = 861, brain, *n* = 2642) based on GTEx project RNA-Seq data. PSI, percent spliced in index. Statistical significance was calculated with unpaired, two-tailed *t* test (*NS* non-significant; *P < 0.05; **P < 0.01 and ***P < 0.001).

Sequences of 3′UTRs are very important in the regulation of mRNA stability, cellular localization and translation efficiency. Based on the analysis of RefSeq data, we could distinguish at least eleven *CELF1* 3′UTR isoforms that could be regulated by alternative splicing and alternative polyadenylation (Fig. 1e). In this study, we showed that the distribution of *CELF1* 3′UTR isoforms is regulated during skeletal muscle, heart and brain development and is significantly different in these tissues (Fig. 2c,d). Therefore, we hypothesized that different *CELF1* 3′UTR isoforms may affect the final levels of CELF1 protein. To test this hypothesis, we first determined whether the lengths and sequences of the *CELF1* 3′UTRs correlated with the translation efficiency in different cell lines. For this purpose, HeLa (epithelial, originate from human cervix), HepG2 (epithelial, originate from human liver) and COS-7 (fibroblasts, originate from African green monkey kidney) cells were transfected with luciferase reporter constructs containing one of three abundant *CELF1* 3′UTR variants: the short isoform *a* (*a1*), which ended upstream of APAS2 (3′UTR-*a1*; approximately 500 nt); the longer isoform *a* (*a2*), which ended upstream of APAS3 (3′UTR-*a2*; ~ 3000 nt); and isoform *d*, which ended upstream of APAS1 (3′UTR-*d*; ~ 2000 nt) (Fig. 1e). After 24 h, the activity of luciferase was measured. Unexpectedly, the effects of the studied 3′UTR variants were significantly different in the various cell lines, and we found no clear correlation between the lengths of *CELF1* 3′UTR isoforms and the protein production (Fig. 4a). The observed differences could be a result of variability in the distribution of miRNAs or RBPs in particular cell lines, which strongly affect mRNA stability and translation efficiency.

In the next step, we investigated whether miRNAs could modulate the levels of different *CELF1* 3′UTR isoforms. In silico prediction revealed several potential binding sites for miRNAs within the *CELF1* 3′UTR (Fig. 1e). Silencing of *DROSHA* and *DGCR8*, two main components of a microprocessor, caused a massive reduction in miRNAs. In cells with miRNA deficiency, the total level of *CELF1* mRNA (Fig. 4b, left panel and Supplementary Fig. S4a, left panel) was not affected, but the level of isoform *a2* was significantly increased, by ca. 20% (Fig. 4b, right panel and Supplementary Fig. S4a, right panel). These data suggest that longer *CELF1* 3′UTR isoform *a2* is under negative control of miRNAs, which are important regulators of *CELF1* expression depending on the distribution of alternative 3′UTRs in mRNA.

As MBNLs and CELF1 play important but opposing roles during skeletal muscle development, we decided to investigate the expression patterns of *CELF1* 3′UTR isoforms in cells with modulated level of MBNL (Fig. 1g) and CELF proteins (Fig. 4c and Supplementary Fig. S4c and d). Silencing and overexpression of *CELF1* induced decreased and increased level of *CELF1* 3′UTR isoform *a*, respectively (Fig. 4c and Supplementary Fig. S4d). On the other hand, MBNL1 showed the opposite effect (Fig. 1g). Furthermore, overexpression of *Celf2* in mouse heart increases the level of 3′UTR isoforms *c* and *d* based on RNA-Seq data^8,31^ (Fig. 4d).

Translation efficiency of *CELF1* mRNA could be affected by both 5′UTR and 3′UTR. Therefore, we compared the level of endogenous Celf1 protein and *Celf1* mRNA in mouse skeletal muscle, heart and brain tissue (Fig. 4e,f). The total level of Celf1 protein was much higher in brain than in striated muscles but the level of mRNA is clearly the lowest in heart and similar in skeletal muscles and brain (Fig. 4f and Supplementary Fig. S4e). These results suggest that the Celf1 protein is synthesized in all types of analyzed tissues, but its level does not depend solely on the level of mRNA. Its steady state level is the highest in tissue with the lowest contribution of mRNA isoforms containing ex5 (Fig. 2a) and 3′UTR variant *a* (Fig. 2b,d). The difference in CELF1 protein level in different tissues is reflected in splicing of CELF1-dependent alternative exons^14^. In tissues with a higher level of CELF1 protein, the CELF1-specific isoforms predominate (Fig. 4g); although, it should be underlined that many RBPs may contribute to splicing profile of analysed alternative exons.

We conclude, that the changes in the length and quality of *CELF1* 5′UTR and 3′UTR could simultaneously affect its translation and in consequence lead to differences in the steady state level of CELF1 in different tissues. We also showed that longer *CELF1* 3′UTR isoform *a* (*a2*) is under stronger pressure of miRNAs than shorter isoform *a* (*a1*). The length and sequence context of the *CELF1* 3′UTR depend on the activity of MBNLs and CELF1 itself, and these factors play the opposite role in this regulation. CELF activity inhibits the production of CELF1 3′UTR isoform *a.* Further investigations are needed to identify these miRNAs and their target sites in the *CELF1* 3′UTR.

## Discussion

CELF1 is an RBP that plays crucial roles during development, tissue differentiation as well as pathological processes. Depending on the presence of binding sites within target RNAs, CELF1 may regulate alternative splicing, mRNA and miRNA degradation pathways or translation efficiency^8,10–13,36^. *CELF1* is expressed in many tissues, especially the heart, skeletal muscles and brain. All *CELF* genes are highly expressed in developing fetal tissues. Interestingly, the level of CELF1 protein is significantly reduced postnatally without changes in the steady state level of *CELF1* mRNA^14^. Changes in the activity of CELF1 protein may be regulated by transcriptional, posttranscriptional or posttranslational mechanisms. For instance, expression of *Celf1* can be controlled at the transcriptional level by activation of one of two promoters in myotubes because it is sensitive to muscle-specific drivers such as myogenin^37^. On the other hand, translation of CELF1 could be repressed by miR-23^38^, which partially explains the significant postnatal reduction in CELF1 levels in skeletal muscles. Regulation of the phosphorylation state of CELF1 is another posttranscriptional regulatory mechanism. Phosphorylation at serine 28 is mediated by AKT serine/threonine kinase (AKT) or Protein Kinase C α/β (PKCα/β), and phosphorylation at serine 302 is mediated by the cyclin D/cdk4/6 signalling cascade^39^. However, hyperphosphorylation of CELF1 causes upregulation of its steady state level and activity in skeletal muscles in the context of DM1^21^.

In this work, we showed that alternative splicing within the 5′ and 3′ UTRs of *CELF1* is another mechanism that controls *CELF1* expression. Significantly different splicing profiles of *CELF1* UTRs characterize various human and mouse tissues, such as skeletal muscles, heart and brain (Fig. 2). Alternative splicing of exons forming the 5′UTR is regulated in similar ways in transcripts produced from two analyzed promoters and depends on the tissue type and/or developmental stage (Fig. 2a,b). One of the exons, ex5, features an alternative AUG codon. Its inclusion has important functional implications. Two CELF1 protein isoforms differing in the lengths of their N-termini can be translated (Fig. 3c). The existence of two CELF1 isoforms in developing embryonic tissues was postulated previously^33^. More recently, it was shown that those two isoforms have different impact on regulation of translation efficiency of *Elavl4* mRNA by binding its 5′UTR during neural development^36^. Our data suggest, that these isoforms have also slightly different splicing activity (Fig. 3f), while they are produced at the same level (Fig. 3e). It also was shown, that changes in subcellular localization of CELF1 has significant impact on muscles physiology^40^. However, this work shows, that two CELF1 protein isoforms have mostly nuclear localization (Fig. 3d). One could also predict that these two isoforms are phosphorylated with different efficiencies or have different affinities for various RNA targets.

Our data also show that the length and sequence context of the *CELF1* 3′UTR could be a result of alternative splicing and alternative polyadenylation (Figs. 1e, 2c). The distribution of different 3′UTR isoforms strongly depends on tissue type and developmental stage. Distinct isoforms could be under the pressure of various miRNAs or RBPs whose concentrations are different in various cell types (Fig. 4). The impact of miR-23 or miR-322/503 on *Celf1* expression was previously described^38,41^; however, our work shows that in certain conditions, mRNA variants can gain or lose binding sites of these miRNAs. For example, in the brain, the main isoform *d* has neither miR-23- nor miR-322/503-sensitive sequences, but in skeletal muscles, miR-23- or miR-322/503-sensitive isoform *a2* or *a1*, respectively, predominates (Fig. 2d,e). The impacts of other miRNAs potentially targeting *CELF1* mRNA should be further examined.

Two RBP families, MBNLs and CELFs, can regulate the expression of several crucial tissue-specific factors at different stages of RNA metabolism, mostly through antagonism^8^. It has been shown that both classes of proteins bind their mRNAs and regulate their stability^42,43^. In this study, we showed that MBNLs can also control the expression of *CELF1* by regulating the distribution of its 5′UTR and 3′UTR isoforms (Fig. 1g and Supplementary Fig. S2). Previous reports showed that knockout of only one paralog of *Mbnl* genes is insufficient to increase Celf1 level. Mice with depletion of Mbnl1^44^ or Mbnl2^45^ manifest some DM-like phenotypes without significant effect on skeletal muscle weakness and heart arrhythmias. Lack of some DM-like symptoms and no change in Celf1 level in *Mbnl1* knockout mouse could be partially explained by the elevated Mbnl2 level in this mouse model^46^. While, a model with a decreased level of both Mbnl1 and Mbnl2 developed severe DM-like muscle defects and reflect much stronger splicing changes, similar to those observed in DM patients^22,46^ suggesting a crucial, cooperative role of insufficiency of both Mbnl1 and Mbnl2 in the DM pathomechanism. We showed, that disruption of alternative *CELF1* ex5 splicing and 3′UTR isoforms in DM1 muscles (Fig. 1) caused by functional inaccessibility of MBNL has a significant impact on CELF1 protein level in the affected tissue. Our findings suggest that an increased level of CELF1 protein in DM1 muscles could be caused not only by hyperphosphorylation of the protein^21^ but also by regulation of an alternative isoforms of *CELF1* UTRs which favor the efficient production of a CELF1 protein.

RBP autoregulation through binding to its RNA is a common mechanism. More recently we presented, that autoregulation of *MBNL1* is driven by skipping of ex1 in *MBNL1* pre-mRNA, giving rise to a non-functional protein^47^. In this work, we showed that *CELF1* can also regulate its expression by modulating its 3′UTR isoform distribution (Fig. 4c). These processes are also regulated by the other family member, CELF2 (Fig. 4d). Both proteins promote the formation of isoforms with proximal 3′UTR sequences with different sensitivities to miRNAs and other RBPs.

In summary, changes in CELF1 protein levels without changes in *CELF1* mRNA levels have been well documented thus far; however, in this work, we showed that both the length and sequence context of *CELF1* 5′UTR and 3′UTR change during ontogenesis, yielding numerous mRNA isoforms with different regulatory potential. Variation in the 5′UTRs directly affects the level of two CELF1 protein isoforms, and they have slightly different splicing activity. Furthermore, changes in the 3′UTR sequence may affect the quantity of CELF1 by altering the regulatory pressure of specific miRNAs and RBPs. Therefore, posttranscriptional modifications of *CELF1* UTR sequences contribute to CELF1 activity under physiological and pathological conditions such as DM1, and modulation of alternative splicing of *CELF1* can be considered as a potential therapeutic strategy.

## Materials and methods

### cDNA from human tissues

cDNA from non-DM and DM1 skeletal muscle samples were generously donated by Dr Charles Thornton (University of Rochester, NY, USA)^48^. cDNA panels from adult tissues (Human MTC Panel I, cat. no. 636742) and fetal tissues (Human Fetal MTC Panel, cat. no. 636747) were purchased from Clontech. According to the company description tissue samples were pooled from the following numbers of individuals: fetal skeletal muscle 13 spontaneously aborted male/female Caucasian fetuses, skeletal muscles 4 male/female Caucasians (ages 25–56), heart 3 male Caucasians (ages 24–41) and brain 8 male Caucasians (ages 43–65). Individual cDNA concentration was normalized using the following genes: alpha-tubulin, beta-actin, G3PDH and phospholipase A2.

### RNA and proteins from mouse tissues

Skeletal muscles (tibialis anterior), hearts and brains were extracted from female C57BL/6J mice in 1, 5, 14 and 90 days after the birth. Mice were euthanized by carbon dioxide inhalation followed by cervical dislocation. All animals were held in the animal facility of the Center of Advanced Technologies, Adam Mickiewicz University, Poznan, Poland (Breeders Register no. 062). The study was approved by the National Ethics Committee for Animal Testing. The study was carried out in compliance with the ARRIVE guidelines. All tissues were obtained according to the National Ethics Committee for Animal Testing and Polish law guidelines and regulations.

### Plasmids

Genetic constructs for expression of CELF1-AUG1 and CELF1-AUG2 were cloned by insertion of *CELF1* sequences into a pEGFP-C1 backbone (Clontech). Both inserts, which were based on murine *Celf1* open reading frames, were cloned between the *Xho*I and *BamH*I restriction sites. To avoid the potential impact of GFP on the CELF1 stability or activity we prepared constructs of CELF1 isoforms fused with the Myc-tag on their C-termini. For this purpose, pcDNA3.1 plasmid (Invitrogen) and inserts were amplified with primers pcDNA_CELF1_F and pcDNA_CELF1_R or CELF1_AUG1, CELF1_AUG2 and CELF1_R, respectively using CloneAmp HiFi PCR Premix (Takara Bio; 639298) and ligated with NEBuilder HiFi DNA Assembly Cloning Kit (New England Biolabs; E5520). To measure transfection and transcription efficiency we cloned a cassette containing an internal ribosome entry site (IRES) and *GFP* open reading frame between *CELF1* open reading frame and pcDNA3.1 polyadenylation signal. This cassette was amplified from MIGR1 plasmid^49^ with IRES_F and IRES_R primers using CloneAmp HiFi PCR Premix (Takara Bio; 639298). pcDNA3.1 plasmid containing *CELF1* open reading frames were amplified with pcDNA_IRES_F and pcDNA_IRES_R primers and ligated with IRES-GFP cassette using NEBuilder HiFi DNA Assembly Cloning Kit (New England Biolabs; E5520). The plasmid for MBNL1 overexpression has been described previously^47^. Plasmids for testing of the protein production of luciferase reporters with different *CELF1* 5′UTRs were generated based on a pmirGLO Dual-Luciferase miRNA Target Expression Vector (Promega, cat. no. E1330). New multiple cloning sites (with *Nde*I, *Sma*I/*Xma*I, and *EcoR*I restriction sites) were cloned between the PGK promoter and the luciferase 2 open reading frame in the *Hind*III restriction site. This plasmid was named 5MCS-pmirGLO. *CELF1* 5′UTR isoform sequences were amplified based on cDNA derived from human skeletal muscle, liver and brain tissues (Human MTC Panel I, Clontech, cat no. 636742). *CELF1* 5′UTR isoforms were cloned between the *Nde*I and *EcoR*I restriction sites. To analyze how the context of the AUG codon impacts CELF1 protein production, two plasmids were created. Two DNA fragments containing 94 bp with an AUG1 or an AUG2 codon were amplified and cloned upstream of luciferase 2 into the 5MCS-pmirGLO plasmid. Both inserts were cloned between the *Nde*I and *EcoR*I restriction sites. Plasmids used to study the translation efficiency of different *CELF1* 3′UTRs were constructed on the backbone of the original pmirGLO Dual-Luciferase miRNA Target Expression Vector. The sequences of *CELF1* 3′UTR isoforms *a1* (473 bp) and *d* (1863 bp) were amplified from cDNA derived from human adult skeletal muscle, and liver tissues (Human MTC Panel I, Clontech, cat. no. 636742), whereas isoform *a2* (2935 bp) was amplified from genomic DNA (isolated from HeLa cells; ATCC, CCL-2). Isoforms *a1* and *d* were cloned between the *Nhe*I and *Xho*I and between the *Nhe*I and *Xba*I restriction sites, respectively. Due to the length of the *a2* isoform, it was amplified in two parts. First, a shorter part (DNA fragment 1; 1196 bp) was amplified and ligated (at the *Pme*I and *Nhe*I restriction sites) into the pmirGLO vector, then a longer part (DNA fragment 2; 1685 bp) was amplified and ligated (at the *Nhe*I and *Xho*I restriction sites) downstream of the first part. *NF1* and *cTNT* splicing minigenes have been described previously^34,35^. CELF1 binds with the sequence of 87 nucleotides of the intronic region upstream to *NF1* alternative exon 23a or the sequence between 17 and 46 nucleotides of the intronic region downstream to *cTNT* alternative exon 5.

### Cell cultures and transfection

HeLa (ATCC, CCL-2) and COS-7 (ATCC, CRL-1651) cells were cultured in DMEM (Lonza) supplemented with 10% fetal bovine serum (FBS, Sigma) and 1% antibiotic–antimycotic (Sigma). HepG2 (ATCC, HB-8065) cells were cultured in EMEM (Lonza) supplemented with 10% FBS (Sigma), 1% MEM Non-Essential Amino Acid Solution (Sigma) and 1% antibiotic–antimycotic (Sigma). The HSkM (Gibco, A11440) cell line was cultured in HAM-F10 medium (Sigma) supplemented with 20% FBS, 1% antibiotic–antimycotic (Sigma), 10 ng/ml Epidermal Growth Factor (EGF, Sigma), 0.4 μg/ml dexamethasone (Sigma) and 25 μg/ml insulin. The cell lines were cultured in an incubator in an atmosphere with proper humidity, 37 °C and 5% CO_2_. Subsets of HSkM and HepG2 cells were transfected with 50 nM control siRNA (siCtrl) duplexes (Future Synthesis). Other HSkM, HeLa and HepG2 cells were cotransfected with 25 nM *MBNL1* siRNA (Future Synthesis) and 25 nM *MBNL2* siRNA (RiboTask) (siMBNL1&2#1 and siMBNL1&2#2) or with 25 nM *DROSHA* siRNA and 25 nM *DGCR8* siRNA (siDROSHA and siDGCR8) duplexes. HepG2 cells were transfected with 50 nM *CELF1* siRNA (siCELF1#1 and siCELF1#2) duplex (Future Synthesis). All siRNA duplexes were delivered into cells using Lipofectamine 2000 or Lipofectamine 3000 (Invitrogen) according to the manufacturer’s protocol. The sequences of all siRNAs are shown in Supplementary Table S2.

All plasmids were delivered into cells with FuGENE HP (Roche) at a ratio of 1 µg of plasmid and 2 µl of FuGENE HP per 2 ml of culture medium. To assess the splicing activity of the CELF1 isoforms, COS-7 cells were seeded onto 12-well plates and cotransfected with 100 ng of Neurofibromin 1 (*NF1*) and cardiac troponin T (*cTNT*) minigenes and various amounts of CELF1-AUG1 or CELF1-AUG2 plasmids (30, 60 or 125 ng). To analyze the impact of MBNL or CELF1 on the distribution of *CELF1* UTR isoforms, HeLa and HepG2 cells were transfected with 1 μg of MBNL1 or CELF1-AUG1 plasmids, respectively, in 6-well plates. For all luciferase plasmids, 250 ng of plasmids were delivered into cells in 48-well plates. HepG2 cells were transfected with i1-luc, i5-luc, AUG1-luc, AUG2-luc and control pmirGLO plasmids, and HeLa, HepG2 and COS-7 cells were transfected with 3′UTR-*a1*, 3′UTR-*a2* and 3′UTR-*d* plasmids. Cells were collected 24 h after transfection.

### Luciferase activity assay

All measurements of luciferase activity were carried out after 24 h of transfection with a Dual-Luciferase Reporter Assay System (Promega, cat. no. E1910) according to the manufacturer’s manual with Infinity 200 PRO (Tecan) equipment. These plasmids have a *Renilla* luciferase open reading frame under a control of different promoter than *Luc2*, which is used as transfection and loading control. *Luc2* activity is shown as a ratio of signal from Firefly and Renilla.

### RNA isolation and RT-PCR

Total RNA was isolated from the tissues or cell lines using TRI Reagent (Sigma) according to the manufacturer’s protocol. 0.5–2 μg of total RNA was used in a reverse transcription reaction with a GoScript Reverse Transcription System (Promega) and random hexamers (Promega) or oligo-dT primers (Promega). All RT-PCRs were performed using GoTaq Flexi DNA Polymerase (Promega). The PCR products were separated on agarose gels with ethidium bromide (Sigma). The gels were visualized on G:BOX (Syngene) equipment, and the DNA band intensities were measured using Gene-Tools software (Syngene). For gels presented in Figs. 1c,f and 2b the unprocessed, full-sized images cannot be provided due to storage server damage. It was not possible to repeat these reactions due to limited amount of patients tissue-derived cDNA; therefore, specificity of primers used in RT-PCR assays were additionally tested for commercially available RNA from human skeletal muscles what is shown in Supplementary Fig. S6. Nevertheless, specificity of all PCR products shown in Figs. 1c,f and 2b were validated by Sanger-sequencing. Real-time PCR was performed using SYBR Green PCR Master Mix (Thermo Fisher Scientific) and a CFX96 Touch Real-Time PCR Detection System (Bio-Rad). *CELF1* expression levels were normalized to the levels of *ACTB* or *GAPDH* as a control. Expression levels of *CELF1* 5′UTR or 3′UTR exons were normalized to the levels of total *CELF1*. Fold changes in expression were calculated using the 2^−ΔΔCT^ method^50^. Localizations of all primers are presented in Fig. 1b, Supplementary Fig. S1.

### Western blot analysis

Proteins were isolated using RIPA buffer (150 mM NaCl, 50 mM Tris–HCl pH 8.0, 1 mM EDTA, 0.5% NP-40, 0.5% Triton X-100, 0.5% sodium deoxycholate, 0.1% SDS) supplemented with SIGMAFAST Protease Inhibitor Cocktail (Sigma). The lysates were sonicated at 4 °C and centrifuged at 18,000×*g* for 10 min at 4 °C. The concentration of protein in mouse samples was measured using the Pierce BCA Protein Assay Kit (Thermo, cat. no. 23225) and 10 µg of protein were loaded pre lane. Before electrophoresis, the samples were denatured at 95 °C for 5 min. The proteins were separated by electrophoresis in a 10% denaturing SDS–polyacrylamide gel and transferred onto a nitrocellulose membrane (Protran BA 85, Whatman) by wet electrotransfer (1 h, 100 V, 4 °C). The membrane was blocked with 5% nonfat milk dissolved in PBS-T buffer (PBS, 0.1% Tween-20) for 1 h. PageRuler Prestained Protein Ladder (ThermoFisher, cat. no. 26617) was used as a molecular mass marker. Incubation with a primary antibody (Supplementary Table S3) was performed at 4 °C overnight. The membrane was washed three times with PBS-T buffer for 10 min. Incubation with a secondary antibody horseradish peroxidase conjugate was performed for 2 h at room temperature. The membrane was washed three times with PBS-T buffer for 10 min. For signal detection, Pierce ECL Plus Western Blotting Substrate (Thermo Scientific) was used. The western blots were visualized on G:BOX (Syngene) equipment, and the protein band intensities were measured using Gene-Tools software (Syngene). Total protein was stained after electrophoresis with SimplyBlue SafeStain (Invitrogen) and visualized on G:BOX (Syngene) equipment.

### RNA-Seq data

Values of percentage splice in index (PSI) of *CELF1* ex4 and ex5 in DM1 skeletal muscles and heart were published previously^31^. The Genotype-Tissue Expression (GTEx) Project was supported by the Common Fund of the Office of the Director of the National Institutes of Health, and by NCI, NHGRI, NHLBI, NIDA, NIMH, and NINDS. The data used for the analyses described in this manuscript were obtained from: the GTEx Portal on 06/01/2020 and/or dbGaP accession number phs000424.v8.p2 on 06/01/2020. Counts of exon-exon junctions in human tissues were provided by GTEx project (file named GTEx_Analysis_2017-06-05_v8_STARv2.5.3a_junctions.gct.gz). Based on these data PSI was calculated according to the following equation, (*CELF1* ex5–ex6 junction counts/(*CELF1* ex5–ex6 junction + *CELF1* ex1–ex6 junction)) × 100. RNA-Seq data sets of human cell lines with *MBNL1* or *MBNL2* knock down and mouse heart with overexpression of *Celf2* were publish previously^8,51–54^. Raw reads were aligned to the Ensembl human (GRCh38) or mouse (GRCm38) genomes with STAR (v2.7)^55^. Aligned reads were counted with featureCounts (v2.0.1)^56^. Alternative splicing events in human cell lines were calculated with rMATS (v4.1)^57^. Level of *Celf1* 3′UTR isoform a in mouse heart after *Celf2* overexpression were calculated as percentage of reads located in this isoform in comparison to all reads located in *Celf1* 3′UTR.

### Statistical analysis

Statistical analysis was performed by an unpaired, two-tailed *t* test using Microsoft Excel (*NS* non-significant; *P < 0.05; **P < 0.01 and ***P < 0.001). Statistical analysis was calculated using the mean from 3–6 biological replicates (*n*) ± standard deviation (SD). Violin plots were generated with *R* (v.4) and ggplot2 package. Exact *p*-values are listed in Supplementary Table S4.

## Supplementary Information

Supplementary Information.
