# Supplementary material for: Distribution of alternative untranslated regions within the mRNA of the CELF1 splicing factor affects its expression
Source: Sci Rep. 2022 Jan 7;12:190. doi: 10.1038/s41598-021-03901-9 (PMC8742084; doi:10.1038/s41598-021-03901-9)
Supplement: Supplementary file 1 — Supplementary Information. [file 41598_2021_3901_MOESM1_ESM.docx]

**Supplementary Information**

**Title:** Distribution of alternative untranslated regions within the mRNA of the *CELF1* splicing factor affects its expression

**Authors:** Arkadiusz Kajdasz^1^, Daria Niewiadomska^1^, Michal Sekrecki^1^, Krzysztof Sobczak^1^

**Affiliation:** ^1^Department of Gene Expression, Institute of Molecular Biology and Biotechnology, Faculty of Biology, Adam Mickiewicz University Poznan, Uniwersytetu Poznanskiego 6, 61-614 Poznan, Poland.

**Corresponding author:** Krzysztof Sobczak, email: ksobczak@amu.edu.pl

**Supplementary Figure S1.** Primer localizations in the 5’UTR and 3’UTR of *CELF1.*


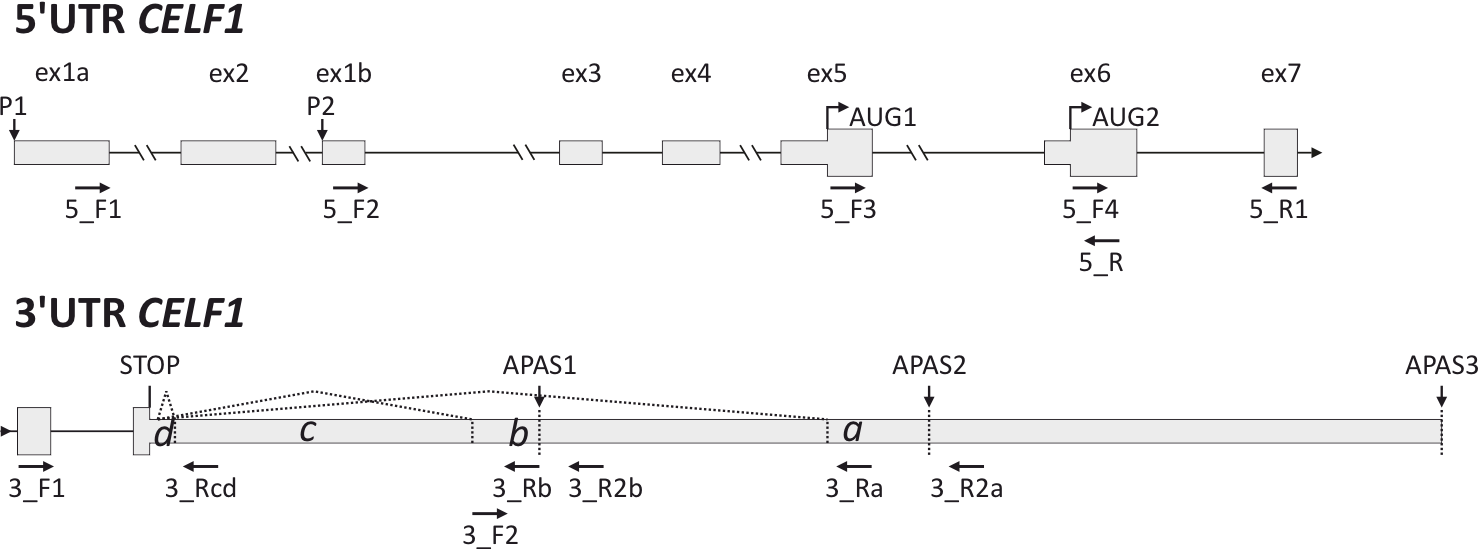


**Supplementary Figure S2.** Impact of MBNLs on CELF1 5’UTR and 3’UTR isoforms distribution.

**(a)** Overexpression of GFP-MBNL1-43 induces the inclusion of *CELF1* ex5 (*n* = 3) and knockdown of MBNL1 and MBNL2 decreases the inclusion of this exon (*n* = 3) (primers 5_F3, 5_F4 and 5_R1). Cropped gels are presented. GFP-MBNL1-43 level after overexpression in HeLa cells was published previously ^1^.

**(b)** *CELF1* 3'UTR isoforms *a* is upregulated after MBNL1 overexpression (primers 3_F1, 3_Ra and 3_Rcd) (*n* = 3). Cropped gels are presented.

**(c)** Levels of MBNL1 and MBNL2 normalised to GAPDH after knockdown with siRNA set#1 in HSkM and HeLa cells. Framed blots are presented.

**(d)** Knockdown of MBNL1 and MBNL2 with other siRNA set decreases the inclusion of *CELF1* ex5 in HepG2 cells (siCtrl, *n* = 6; siMBNL1&2#2, *n* = 5) (primers 5_F3, 5_F4 and 5_R1). *CELF1* 3'UTR isoforms *a* is downregulated after MBNL1 and MBNL2 knockdown with other siRNA set (primers 3_F1, 3_Ra and 3_Rcd) (siCtrl, *n* = 6; siMBNL1&2#2, *n* = 4). CELF1 protein is upregulated after MBNLs knockdown with second siRNA set in HepG2 cells (siCtrl, *n* = 3; siMBNL1&2#2, *n* = 6); Cropped gels are presented.

**(e)** Levels of MBNL1 and MBNL2 normalised to Vinculin after knockdown with siRNA set#2 in HepG2 cells. Framed blots are presented.

Statistical significance was calculated with unpaired, two-tailed *t*-test (NS – non-significant; * *P* < 0.05; ** *P* < 0.01 and *** *P* < 0.001).


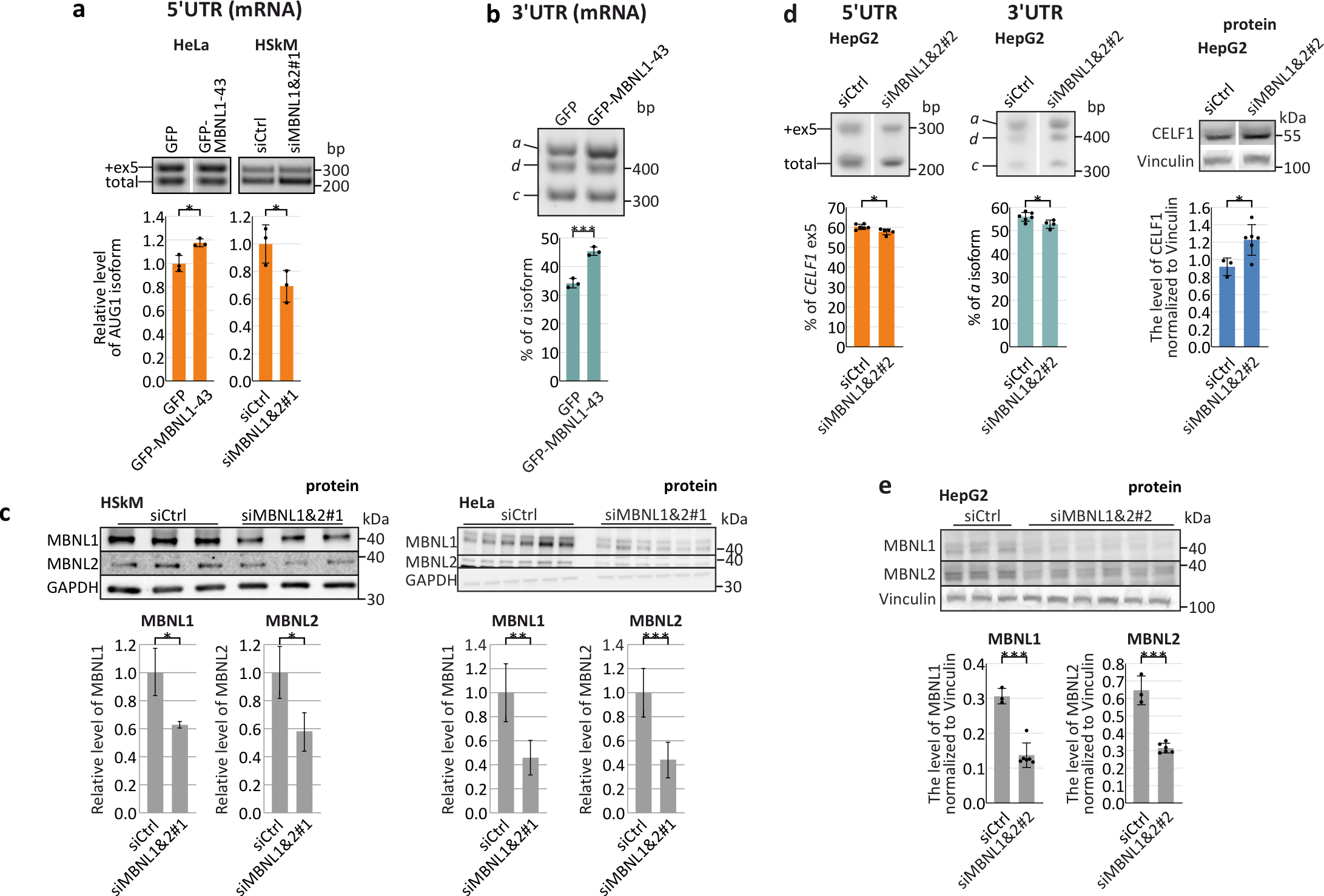


**Supplementary Figure S3.** Profiles of *CELF1* 3’UTR isoforms.

**(a)** Effects of elevated level of MBNL1 and CELF1 on the translation activity of mRNA with *CELF1* 5'UTR isoforms.

**(b)** Overexpression of CELF1-AUG1-Myc and CELF1-AUG2-Myc in COS-7 cells.

Statistical significance was calculated with unpaired, two-tailed *t*-test (NS – non-significant; * *P* < 0.05; ** *P* < 0.01 and *** *P* < 0.001).

**
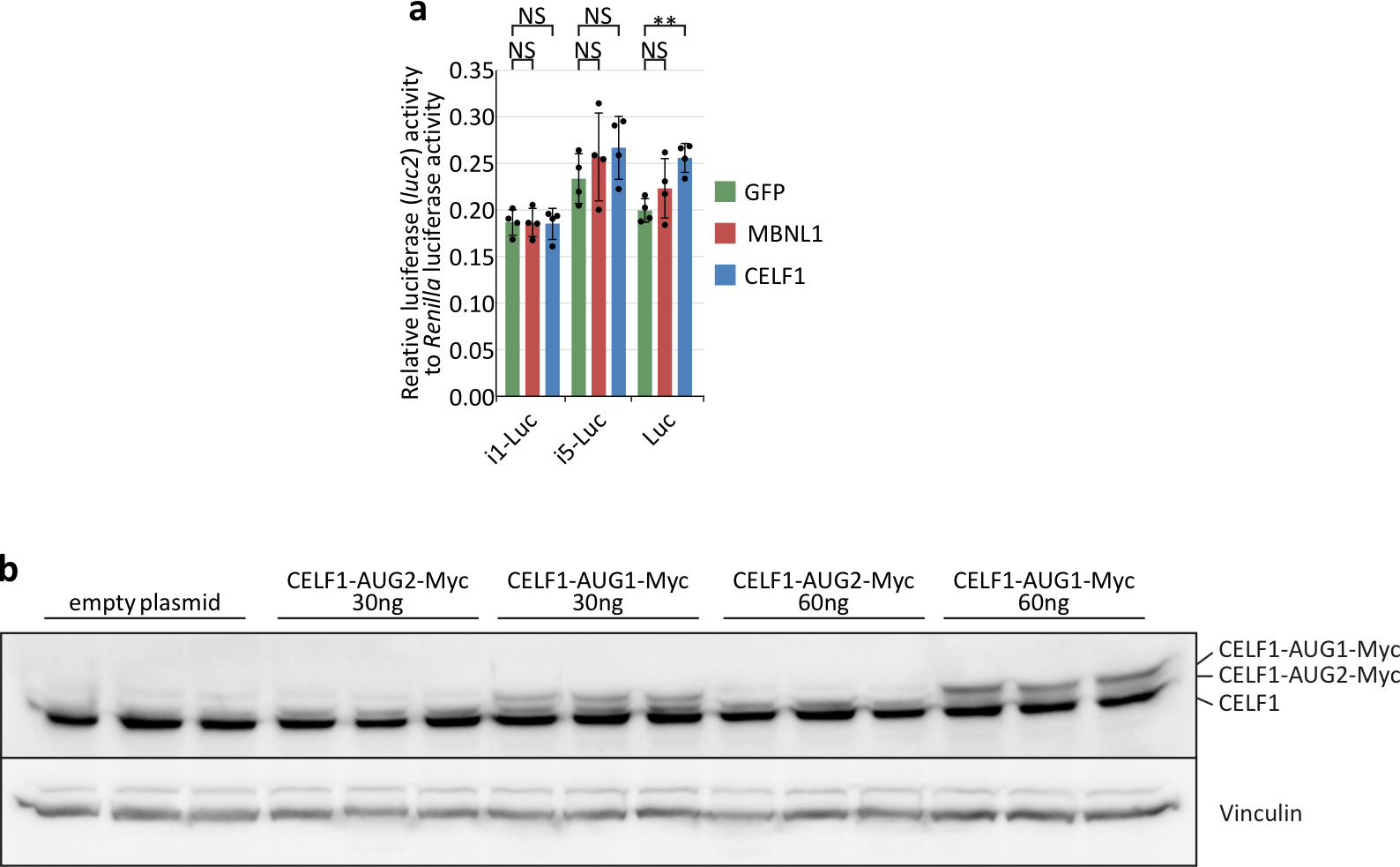
**

**Supplementary Figure S4.** Levels of *CELF1* 3’UTR isoforms under miRNA deficiency conditions.

**(a)** Real-time RT-PCR for *CELF1* mRNA level (normalised to *GAPDH*) and RT-PCR for *CELF1* 3’UTR isoform *a* level (normalised to *CELF1*) (primers 3_F1 and 3_Ra) in HSkM cells after *DROSHA* and *DGCR8* knockdown (*n* = 3). A cropped gel is presented on the right panel.

**(b)** Overexpression of GFP-CELF1 in HepG2 cells. Framed blots are presented.

**(c)** Level of CELF1 normalised to Vinculin after knockdown with siRNA set#1 in HepG2 cells (*n* = 6). Framed blots are presented.

**(d)** The distributions of *CELF1* 3'UTR isoforms are sensitive to CELF1 activity after siRNA set#2 (primers 3_F1, 3_Ra and 3_Rcd) (siCtrl, *n* = 6; siCELF1#2, *n* = 5). Level of CELF1 normalised to Vinculin after knockdown with siRNA set#2 in HepG2 cells (siCtrl, *n* = 6; siCELF1#2, *n* = 4). Cropped gels are presented.

**(e)** Ct values of *Celf1* cDNA after amplification with RT-qPCR in mouse tissues. To cDNA synthesis were used 500 ng of total RNA.

Statistical significance was calculated with unpaired, two-tailed *t*-test (NS – non-significant; * *P* < 0.05; ** *P* < 0.01 and *** *P* < 0.001).


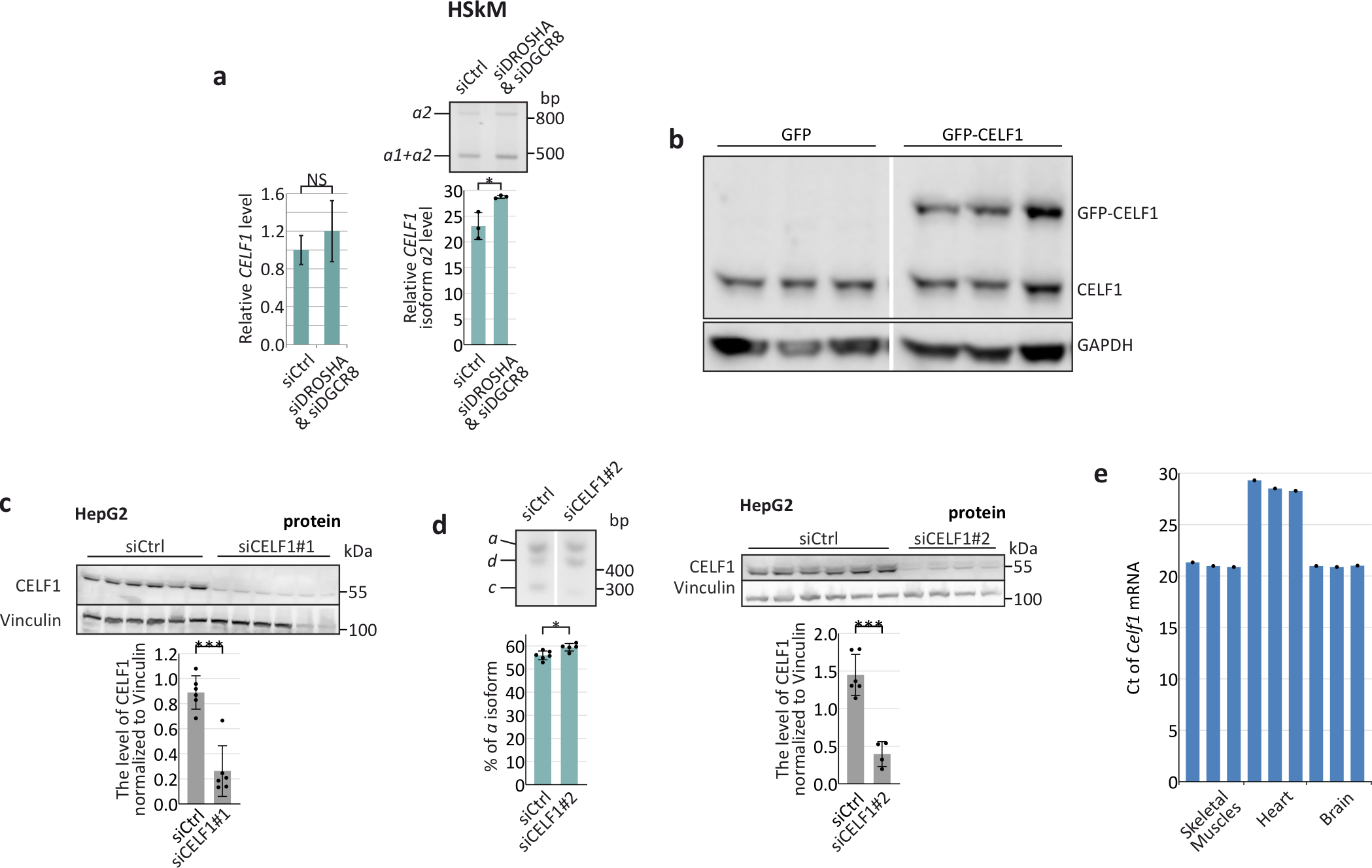


**Supplementary Figure S6.** Unprocessed gels and blots.


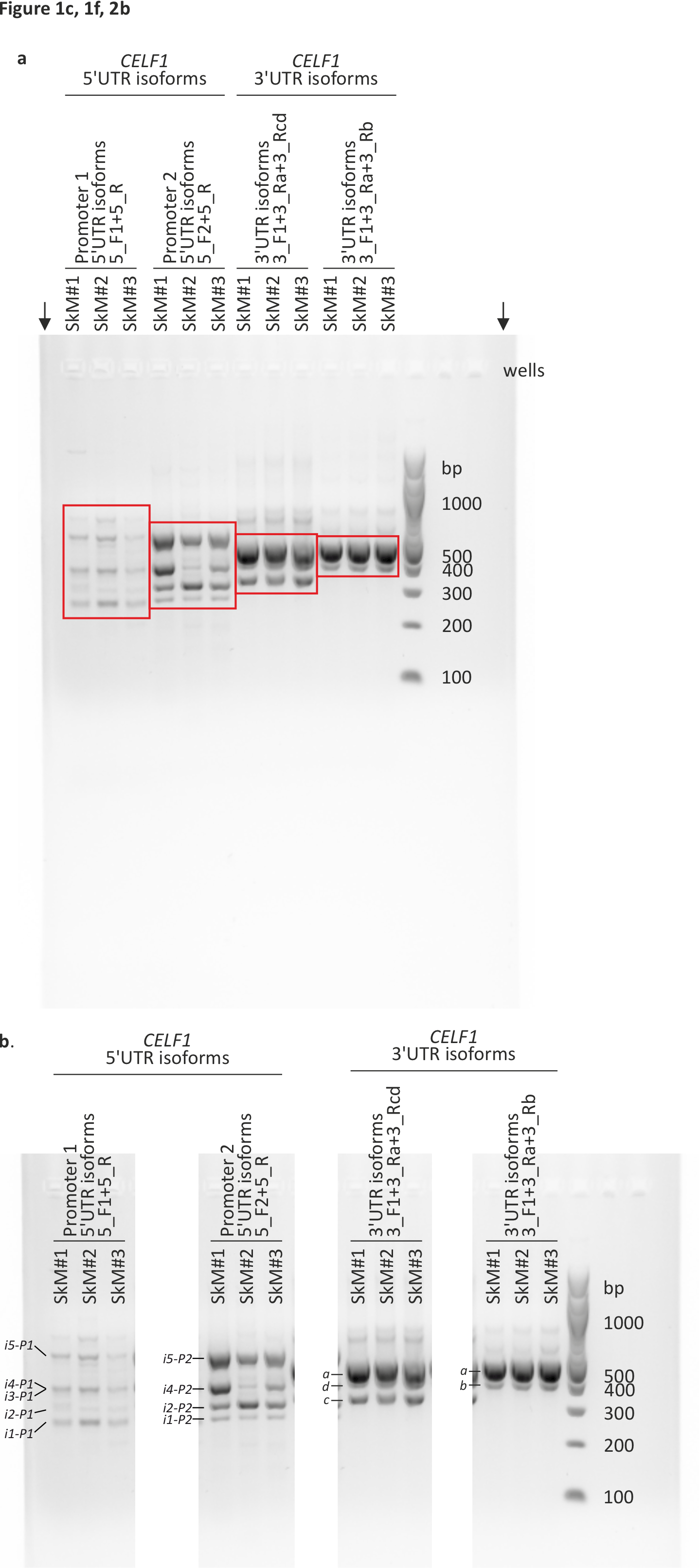


**Fig. SI. Specificity of primers used in the reactions presented in Fig. 1c, 1f and 2b in human skeletal muscles.**

For gels presented in Figures 1c, 1f and 2b the unprocessed, full-sized images cannot be provided due to storage server damage. It was not possible to repeat these reactions due to limited amount of patients cDNA, therefore primers specificity in human skeletal muscles is shown.

**(a)** Gel, specificity of primers used to amplification of *CELF1* 5’UTR isoforms transcribed from the promoter 1 (5_F1 and 5_R) corresponding to Fig. 1c, left panel and 2b, upper panel; specificity of primers used to amplification of *CELF1* 5’UTR isoforms transcribed from the promoter 2 (5_F2 and 5_R) corresponding to Fig. 1c, right panel and 2b, lower panel; specificity of primers used to amplification of *CELF1* 3’UTR isoforms *a*, *d* and *c* (3_F1, 3_Ra and 3_Rcd) corresponding to Fig. 1f, left panel; specificity of primers used to amplification of *CELF1* 3’UTR isoforms *a*, *d* and *c* (3_F1, 3_Ra and 3_Rcd) corresponding to Fig. 1f, right panel.

**(b)** The same gel which is presented in (a). Gel is cropped to indicate *CELF1* UTRs isoforms.

Expected products sizes:

primers 5_F1 + 5_R: *i1-P1*, 273 bp; *i2-P1*, 313 bp; *i3-P1*, 385 bp; *i4-P1*, 384 bp; *i5-P1*, 537 bp;

primers 5_F2 + R_5: *i1-P2*, 288 bp; *i2-P2*, 328 bp; *i3-P2*, 400 bp; *i5-P2*, 552 bp;

primers 3_F1 + 3_Ra: isoform *a*, 475 bp;

primers 3_F1 + 3_Rcd: isoform *c*, 335 bp; isoform *d* 423 bp;

primers 3_F1 + 3_Rb: isoform *b*, 393 bp.

All PCR products were sequenced to confirm their sequences.

Gel edges are indicated with arrows. Wells are indicated. SkM, human skeletal muscles in three biological replicates. Total RNA was obtained from the undefined skeletal muscles (BioChain Inc.). Red rectangle, similar areas from the original gels were used to make the manuscript figures.


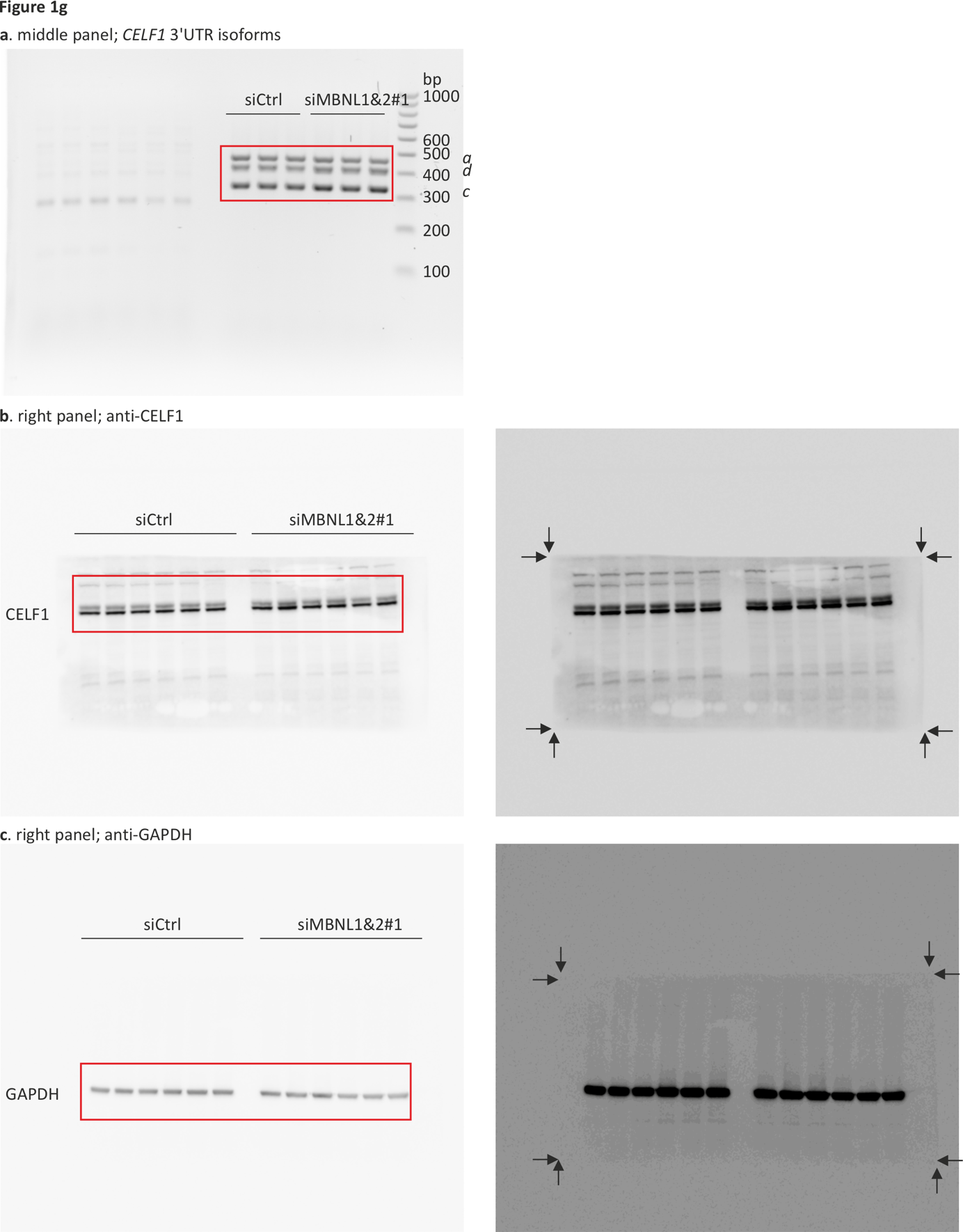


**Fig. SII. Full size images of the blot corresponding to Fig. 1g.**

(a) Gel, *CELF1* 3’UTR isoforms amplification after *MBNL1* and *MBNL2* knock-down with siRNA set #1.

(b, c) Western blots, CELF1 (b) and GAPDH (c) as loading control after *MBNL1* and *MBNL2* knock-down with siRNA set #1. Original images (left panel) and high contrasted images (right panel) are presented; membrane edges are indicated with arrows.

Red rectangle, bands used to make the manuscript figures.


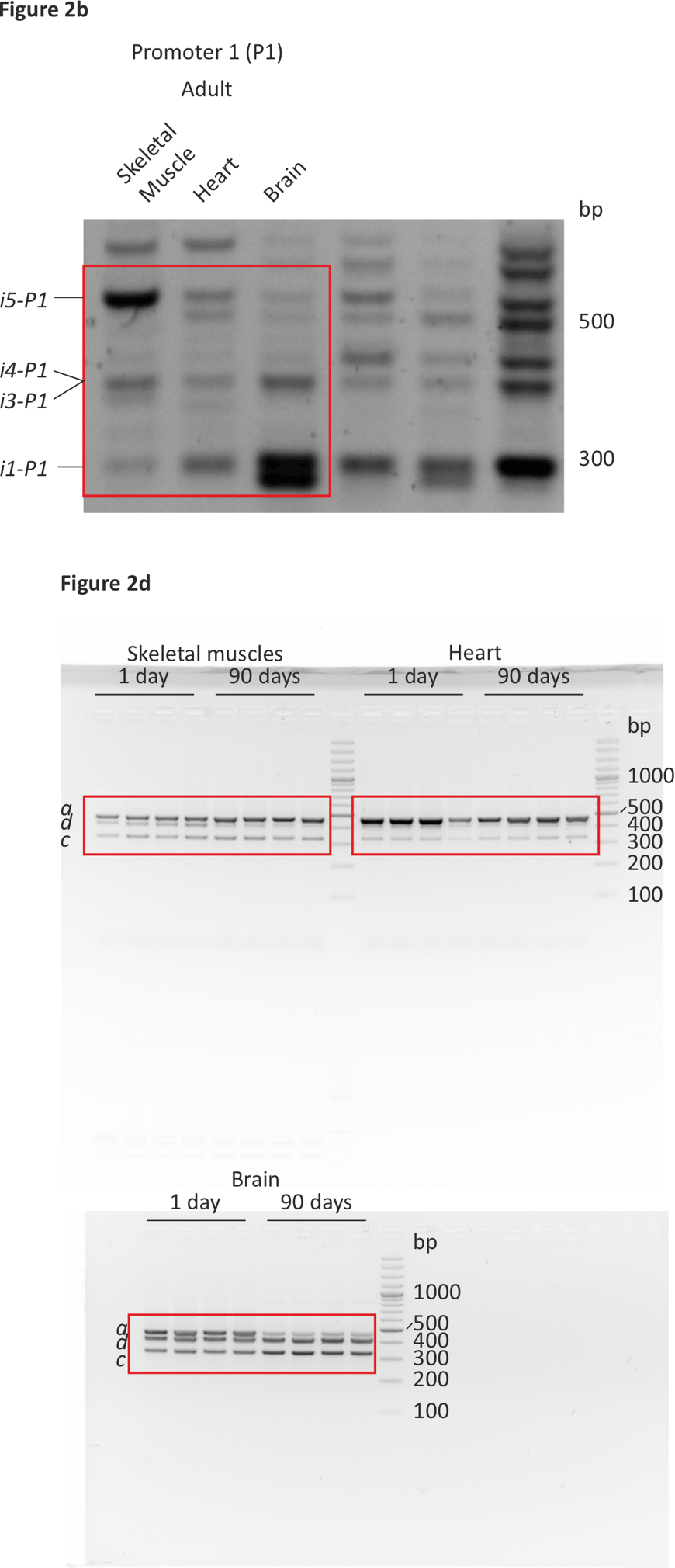


**Fig. SIII. Full size images of the blot corresponding to Fig. 2b and 2d.**

(Upper panel) Gel, *CELF1* 5’UTR isoforms amplification in human tissues.

(Middle and lower panels) Gels, *Celf1* 3’UTR isoforms amplification in mouse tissues.

Red rectangle, bands used to make the manuscript figures.


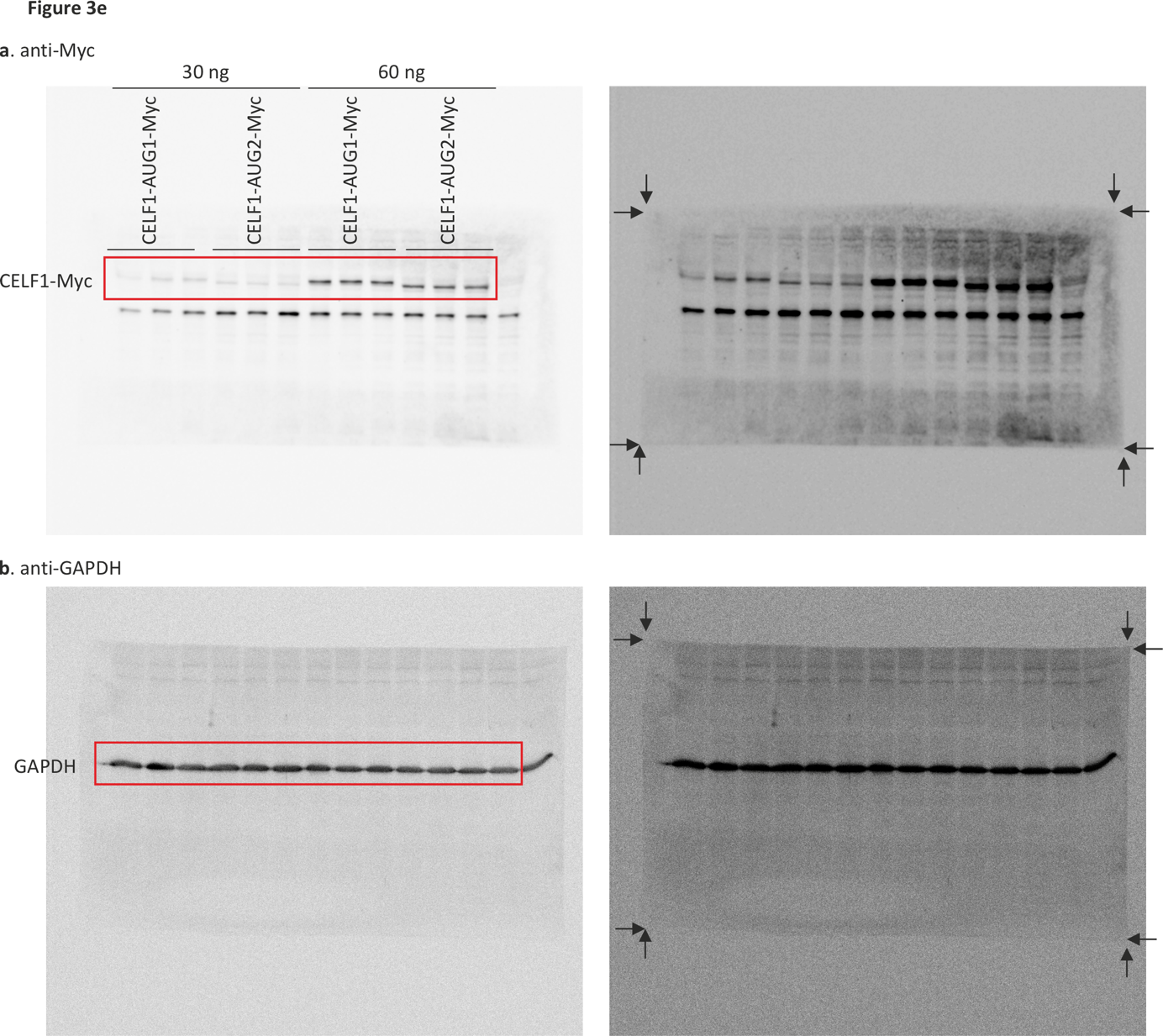


**Fig. SIV. Full size images of the blot corresponding to Fig. 3e.**

(a) Western blot, CELF1-Myc.

(b) Western blots, GAPDH as loading control.

Original images (left panel) and high contrasted images (right panel) are presented; membrane edges are indicated with arrows. Red rectangle, bands used to make the manuscript figures.


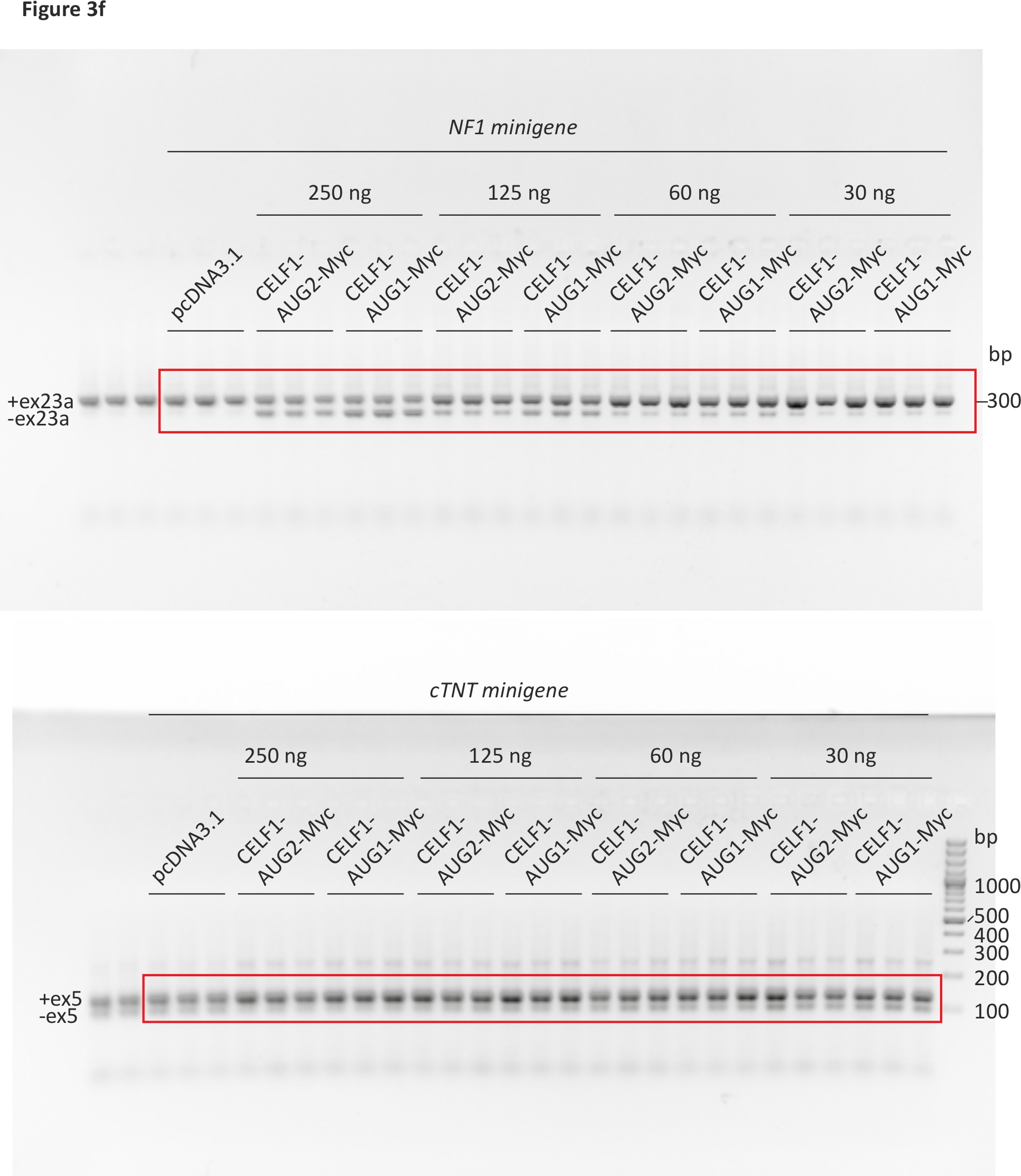


**Fig. SV. Full size images of the blot corresponding to Fig. 3f.**

(a) Gels, alternative splicing of NF1 ex23a (upper panel) and cTNT ex5 (lower panel) after CELF1-AUG1-Myc and CELF1-AUG2-Myc overexpression

Red rectangle, bands used to make the manuscript figures.


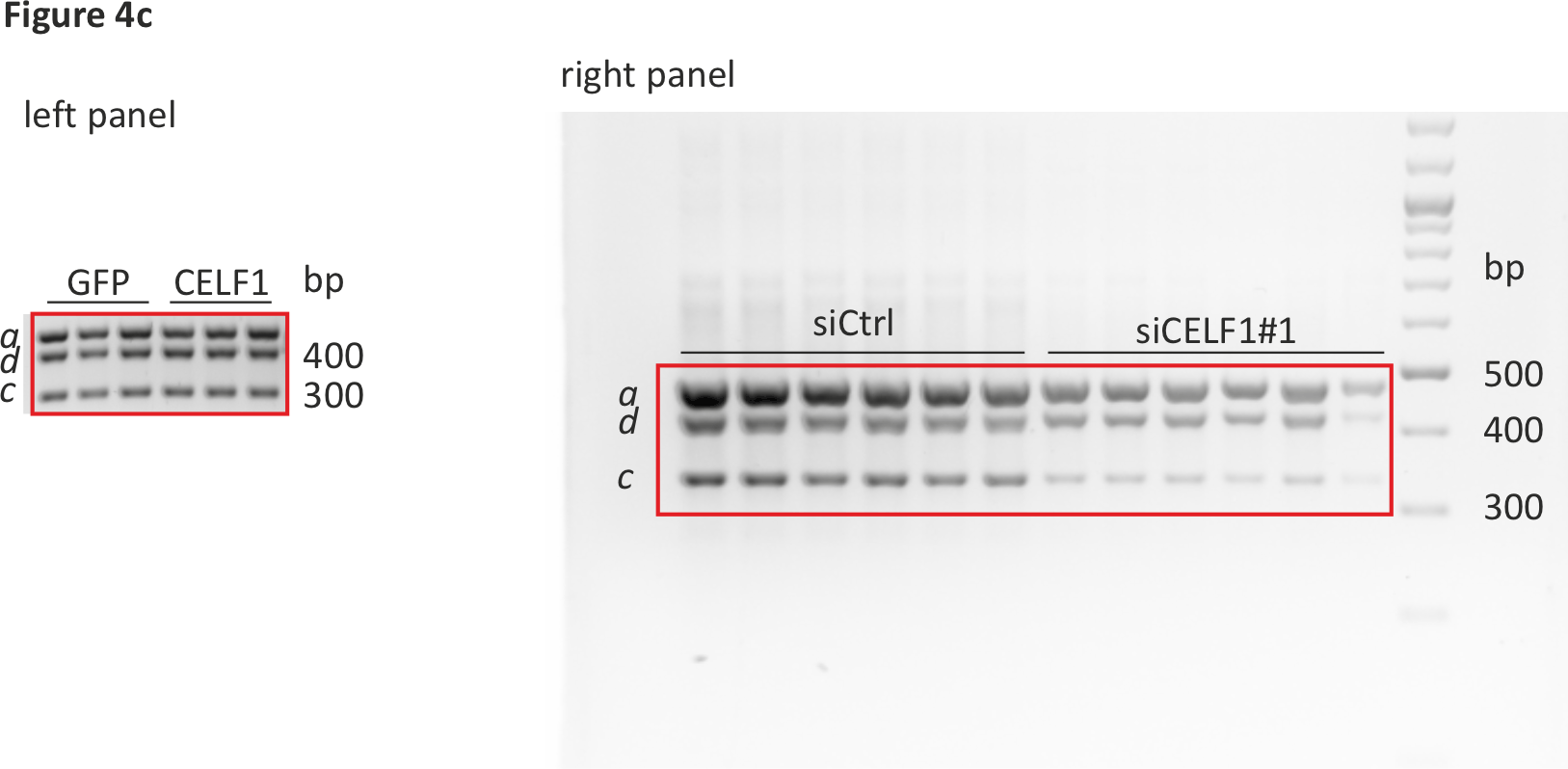


**Fig. SVI. Full size images of the blot corresponding to Fig. 4c.**

Gels, *CELF1* 3’UTR isoforms amplification after GFP-CELF1 overexpression (left panel) and *CELF1* knock-down with siRNA set #1 (right panel). Red rectangle, bands used to make the manuscript figures.


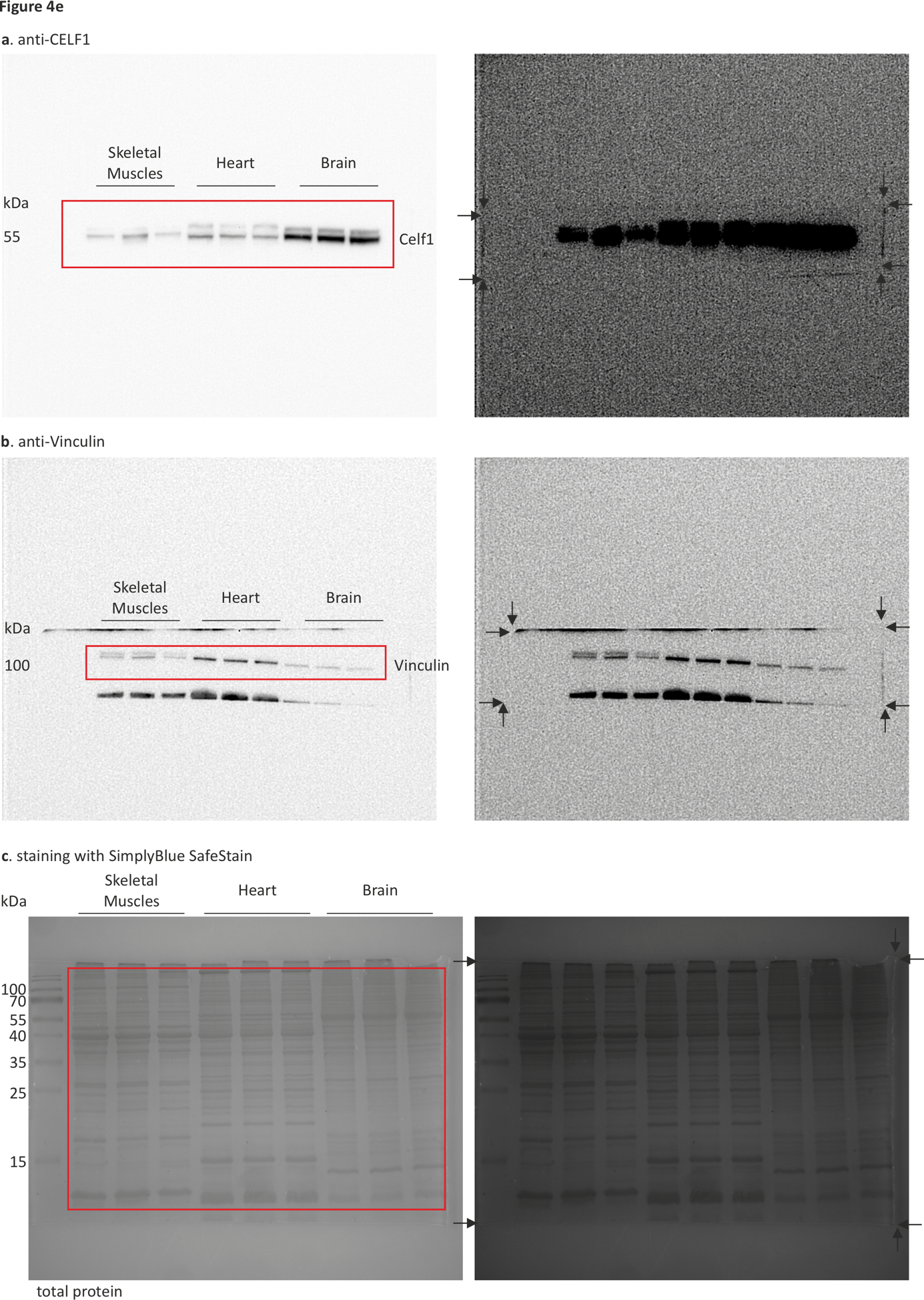


**Fig. SVII. Full size images of the blot corresponding to Fig. 4e.**

(a, b) Western blots, Celf1 (b) and Vinculin (c) as loading control in mouse tissues.

(c) Gel, total protein from mouse tissues stained with SimplyBlue SafeStain.

Original images (left panel) and high contrasted images (right panel) are presented; membrane or gel edges are indicated with arrows. Red rectangle, bands used to make the manuscript figures.


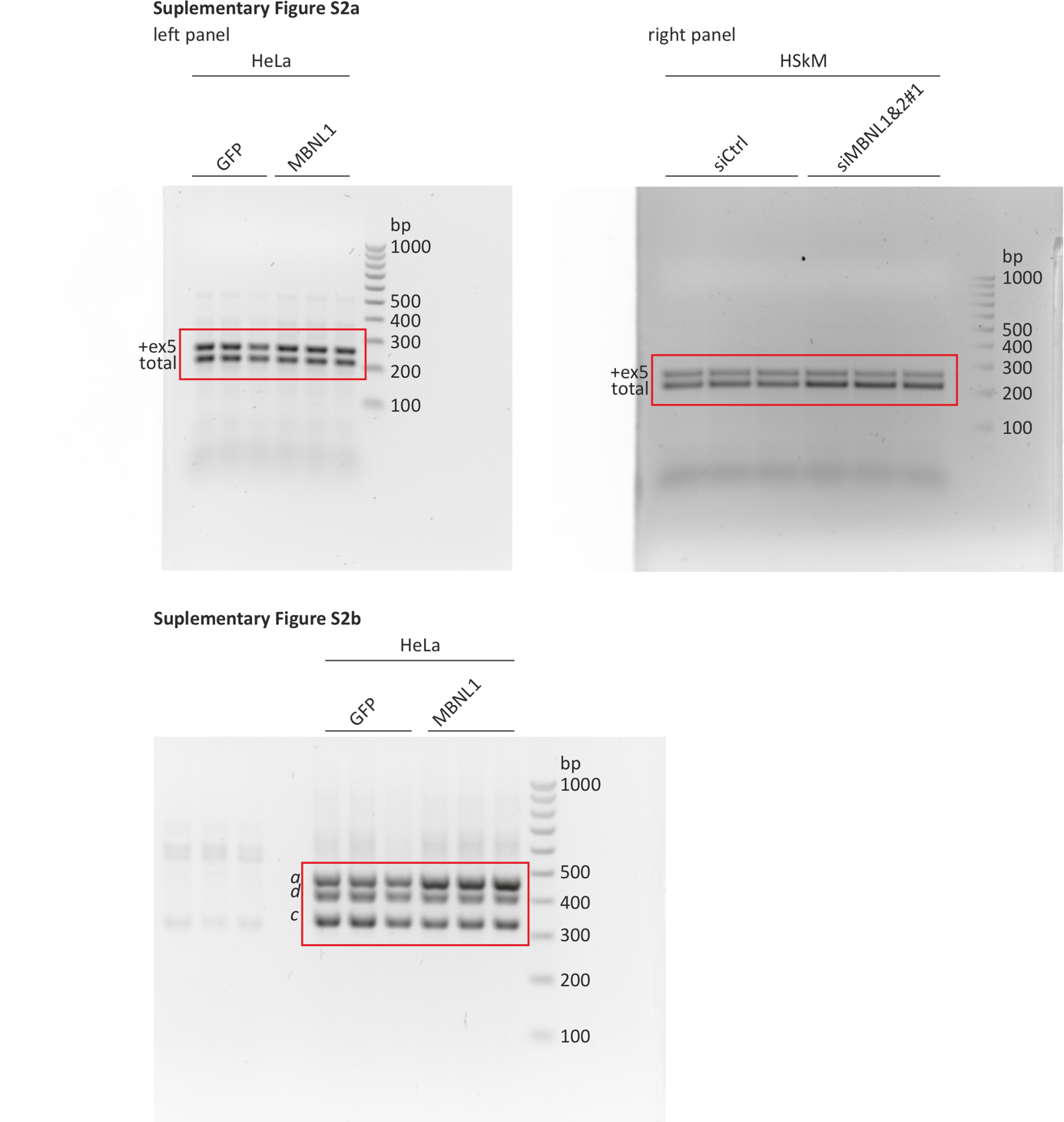


**Fig. SVIII. Full size images of the blot corresponding to Supplementary Fig S2a and S2b.**

Gels, *CELF1* 5’UTR isoforms amplification after GFP-MBNL1 overexpression (left, upper panel) and *MBNL1* and *MBNL2* knock-down with siRNA set #1 (right, upper panel) and *CELF1* 3’UTR isoforms amplification after GFP-MBNL1 overexpression (lower panel). Red rectangle, bands used to make the manuscript figures.


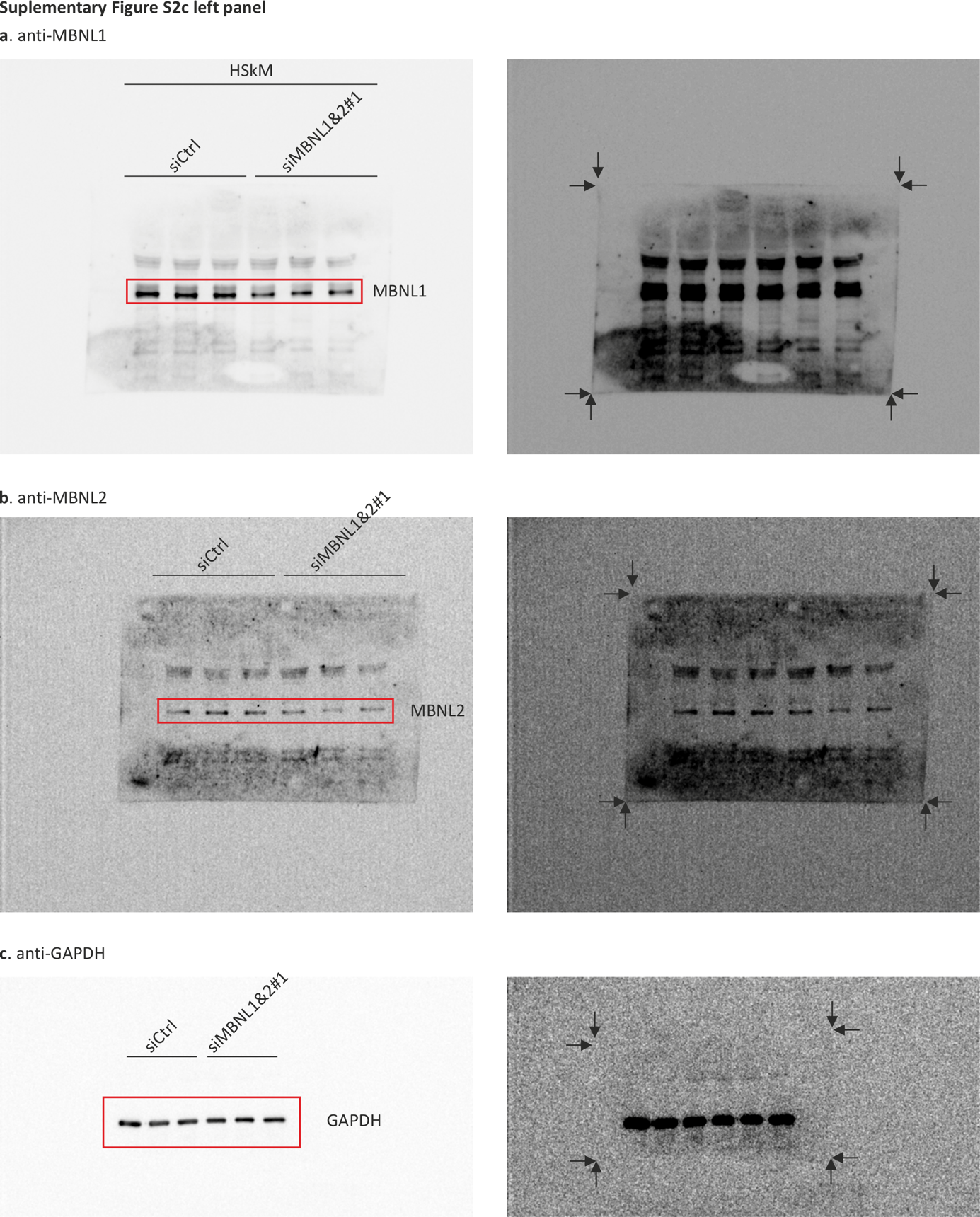


**Fig. SIX. Full size images of the blot corresponding to Supplementary Fig. S2c left panel.**

Western blots, MBNL1 (a), MBML2 (b) and GAPDH (c) as loading control after *MBNL1* and *MBNL2* knock-down with siRNA set #1 in HSkM cells. Original images (left panel) and high contrasted images (right panel) are presented; membrane edges are indicated with arrows. Red rectangle, bands used to make the manuscript figures.


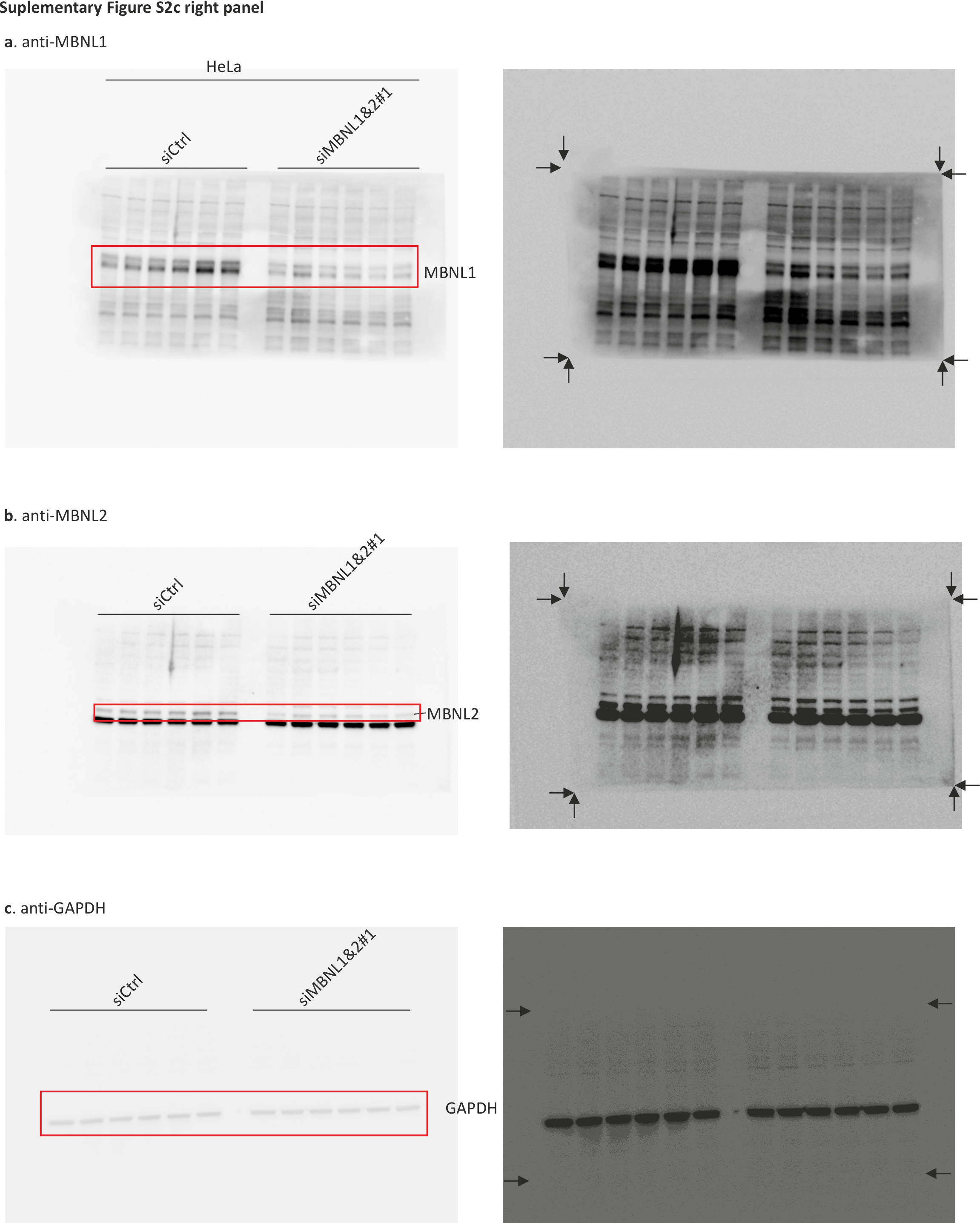


**Fig. SX. Full size images of the blot corresponding to Supplementary Fig. S2c right panel.**

Western blots, MBNL1 (a), MBML2 (b) and GAPDH (c) as loading control after *MBNL1* and *MBNL2* knock-down with siRNA set #1 in HeLa cells. Original images (left panel) and high contrasted images (right panel) are presented; membrane edges are indicated with arrows. Red rectangle, bands used to make the manuscript figures.


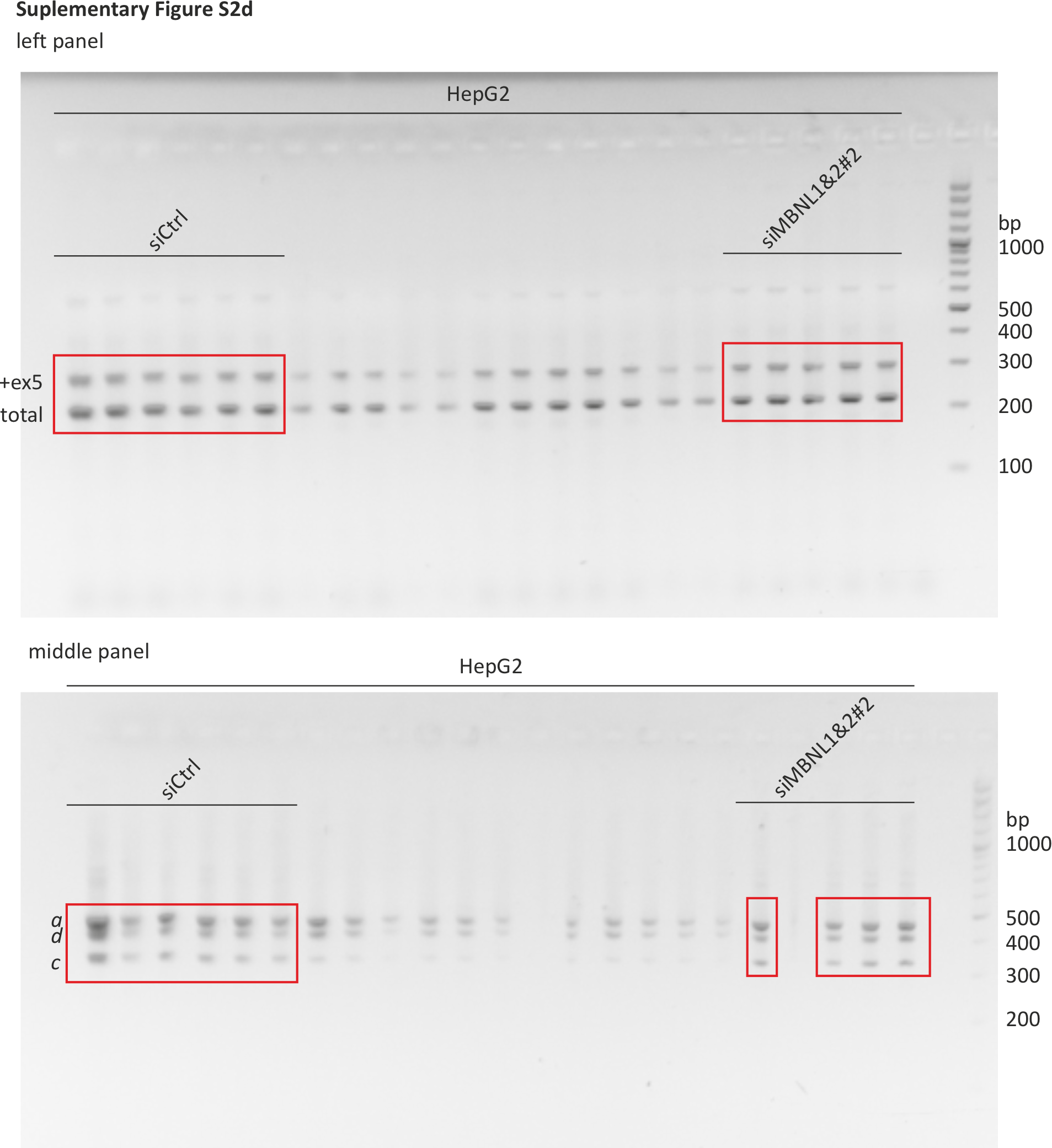


**Fig. SXI. Full size images of the blot corresponding to Supplementary Fig S2d left and middle panels.**

Gels, *CELF1* 5’UTR and 3’UTR isoforms amplification after *MBNL1* and *MBNL2* knock-down with siRNA set #2 in HepG2 cells. Red rectangle, bands used to make the manuscript figures.


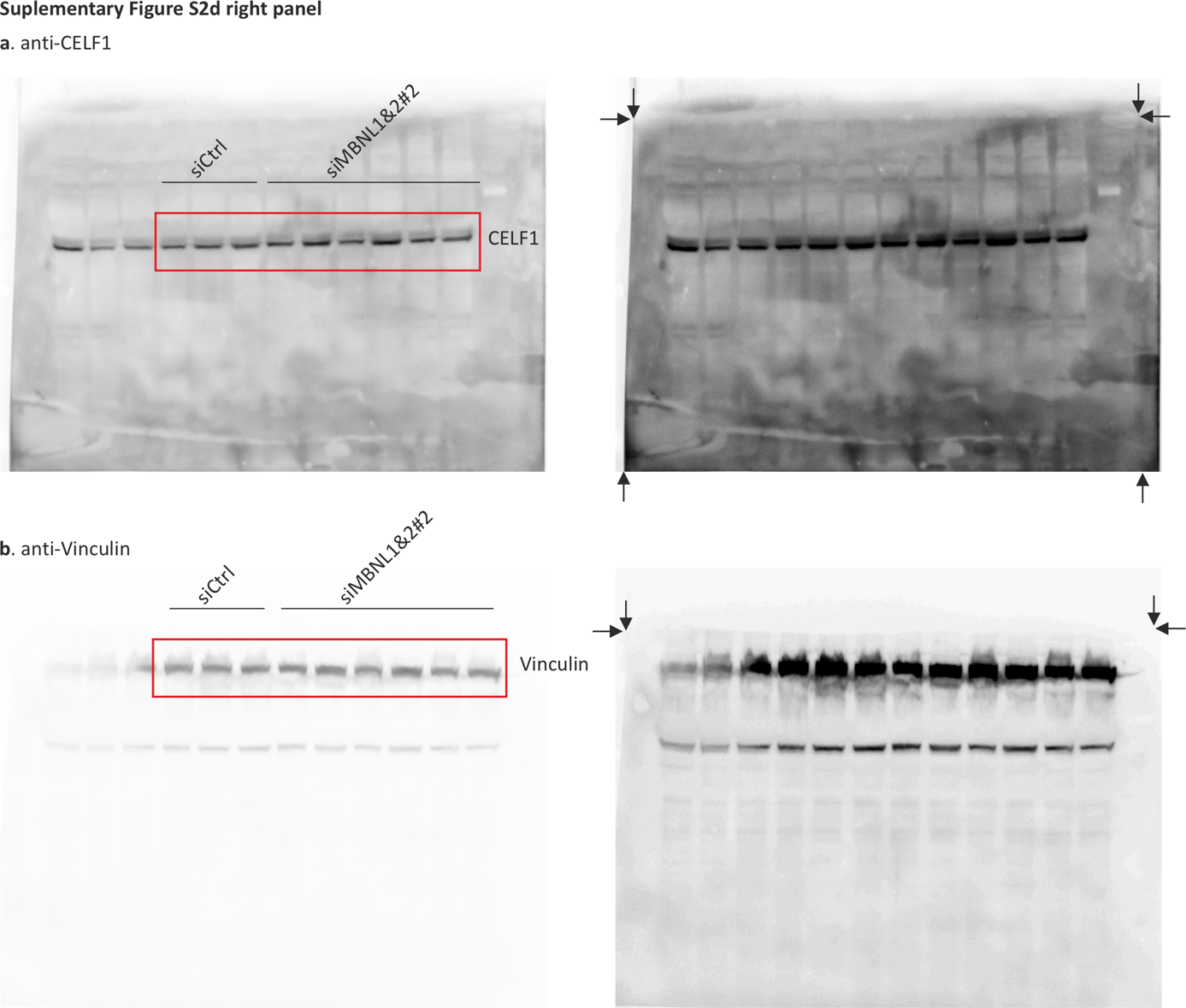


**Fig. SXII. Full size images of the blot corresponding to Supplementary Fig. S2d right panel.**

Western blots, CELF1 and Vinculin (d) as loading control after *MBNL1* and *MBNL2* knock-down with siRNA set #2 in HepG2 cells. Original images (left panel) and high contrasted images (right panel) are presented; membrane edges are indicated with arrows. Red rectangle, bands used to make the manuscript figures.


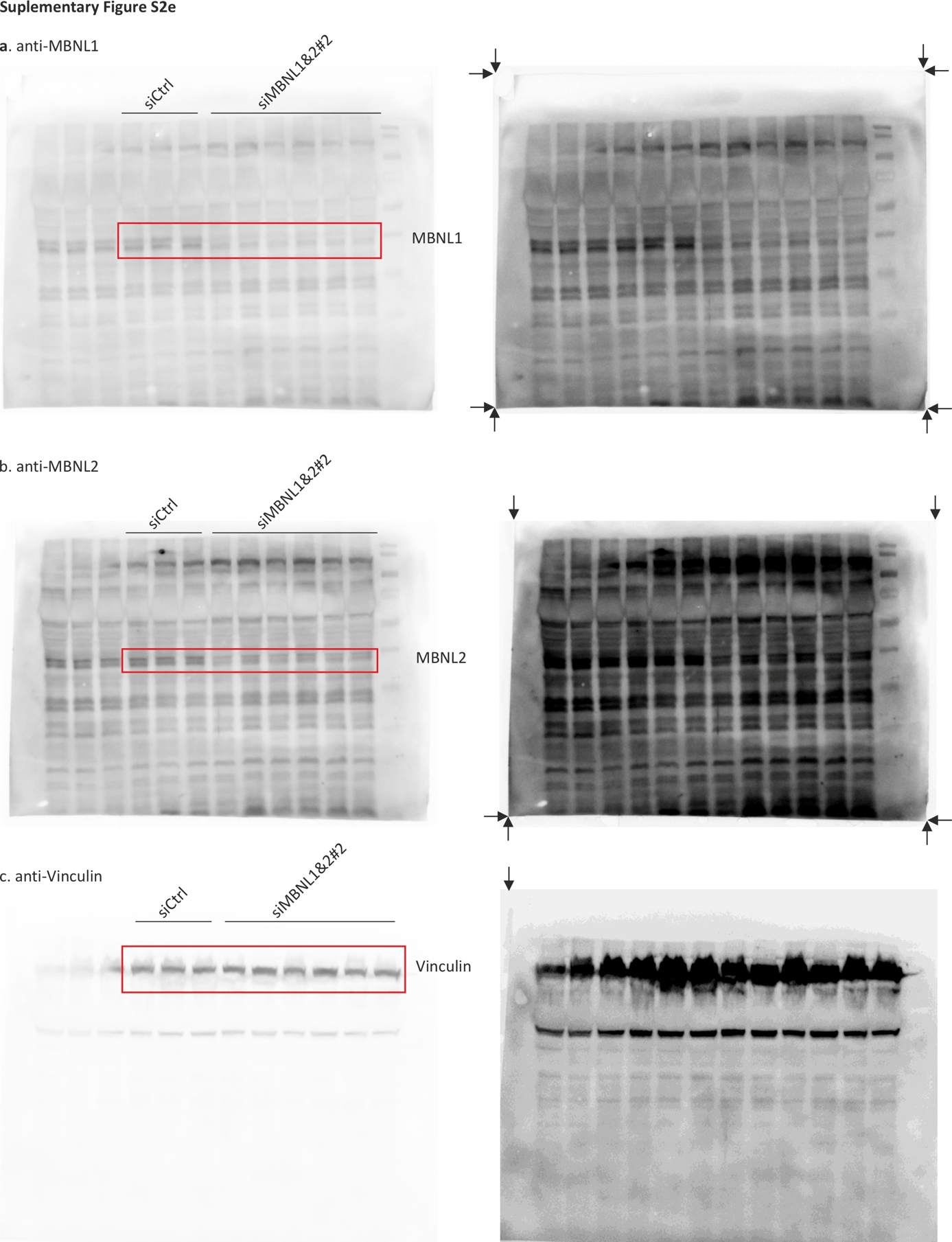


**Fig. SXIII. Full size images of the blot corresponding to Supplementary Fig. S2e.**

Western blots, MBNL1 (a), MBML2 (b) and Vinculin (c) as loading control after *MBNL1* and *MBNL2* knock-down with siRNA set #2 in HepG2 cells. Original images (left panel) and high contrasted images (right panel) are presented; membrane edges are indicated with arrows. Red rectangle, bands used to make the manuscript figures.


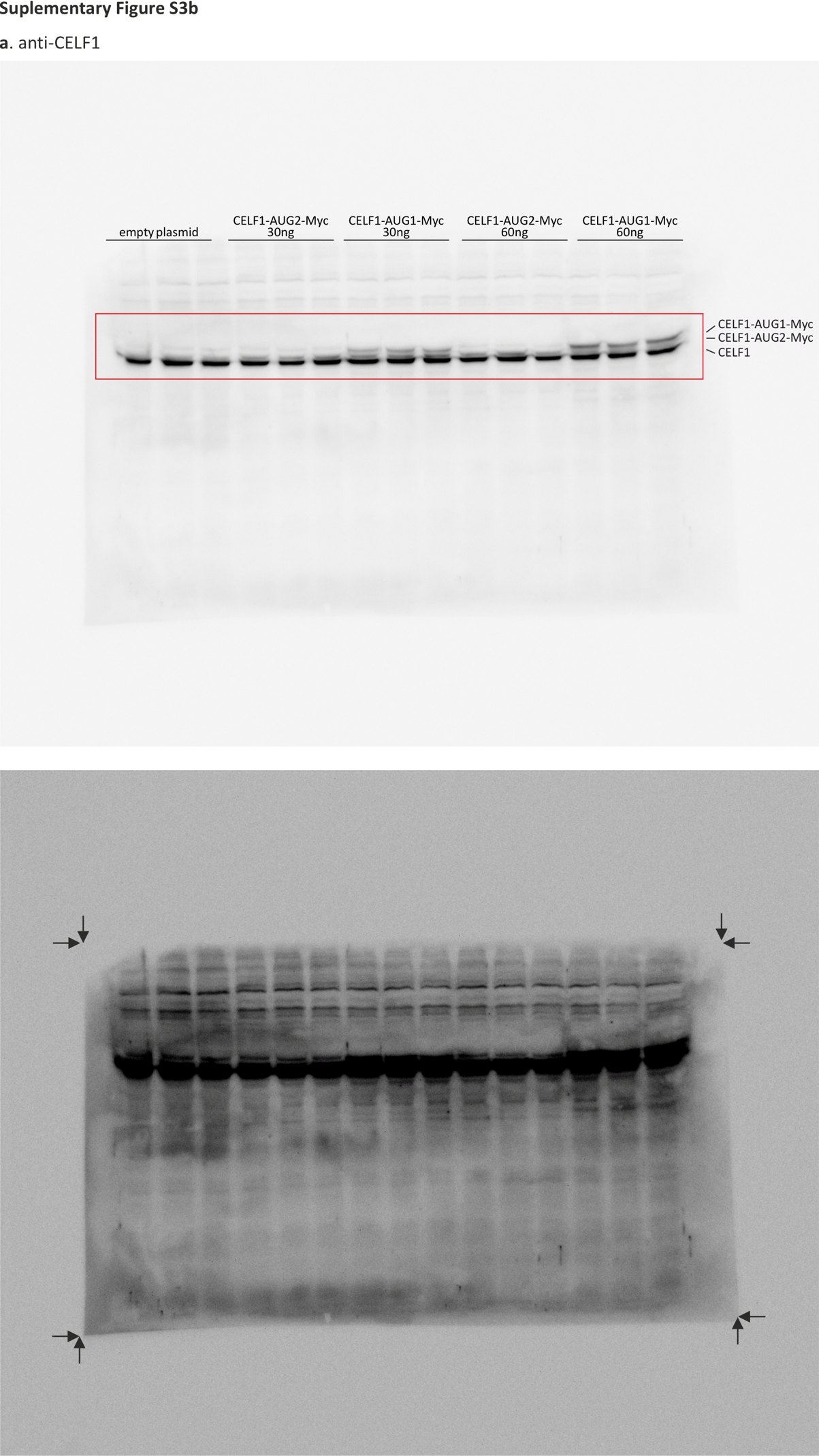


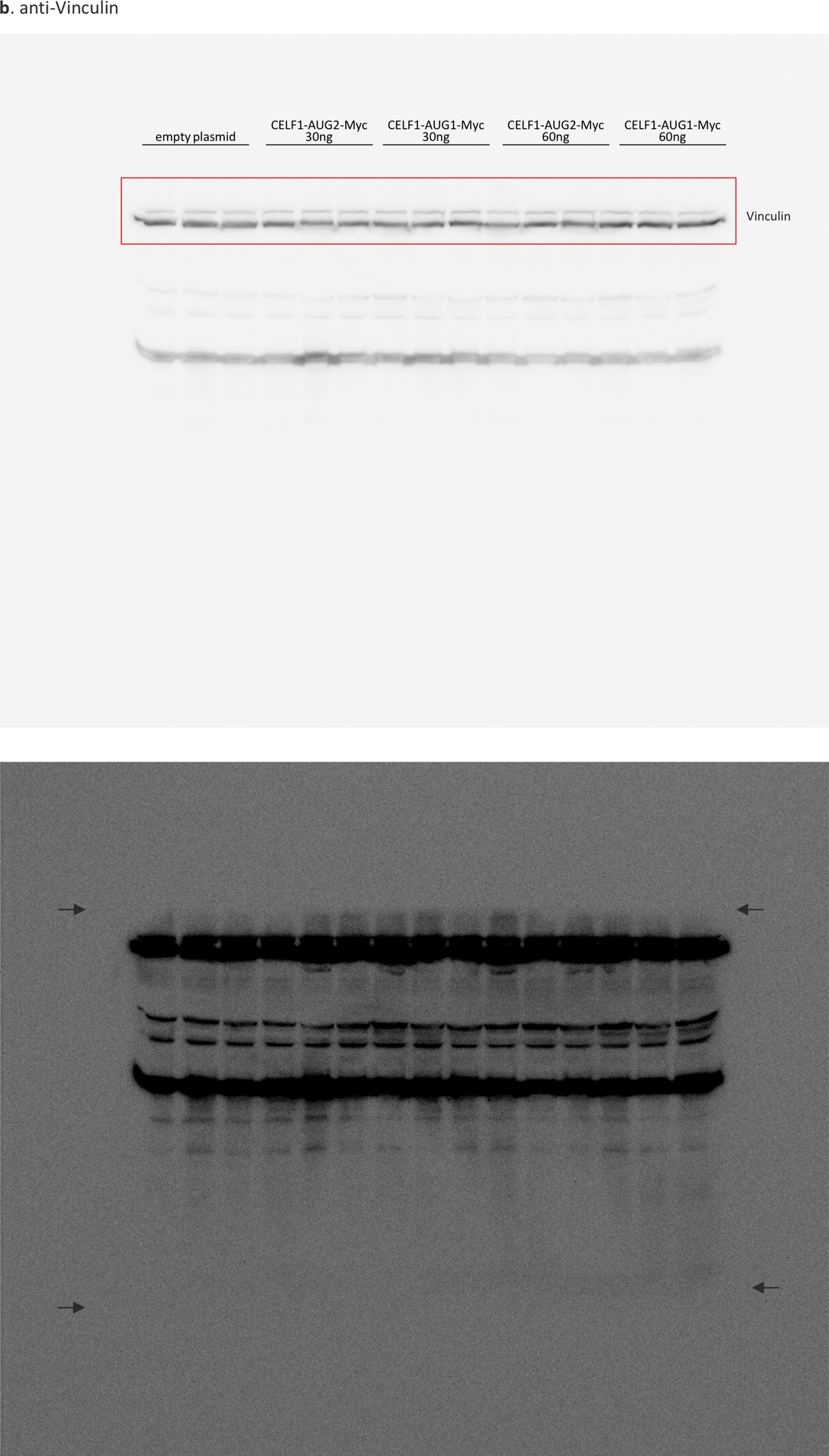


**Fig. SXIV. Full size images of the blot corresponding to Supplementary Fig. S3b.**

Western blots with anti-CELF1 antibody (a) and anti-Vinculin (b) as loading control. Original images (upper panels) and high contrasted images (lower panels) are presented; membrane edges are indicated with arrows. Red rectangle, bands used to make the manuscript figures.


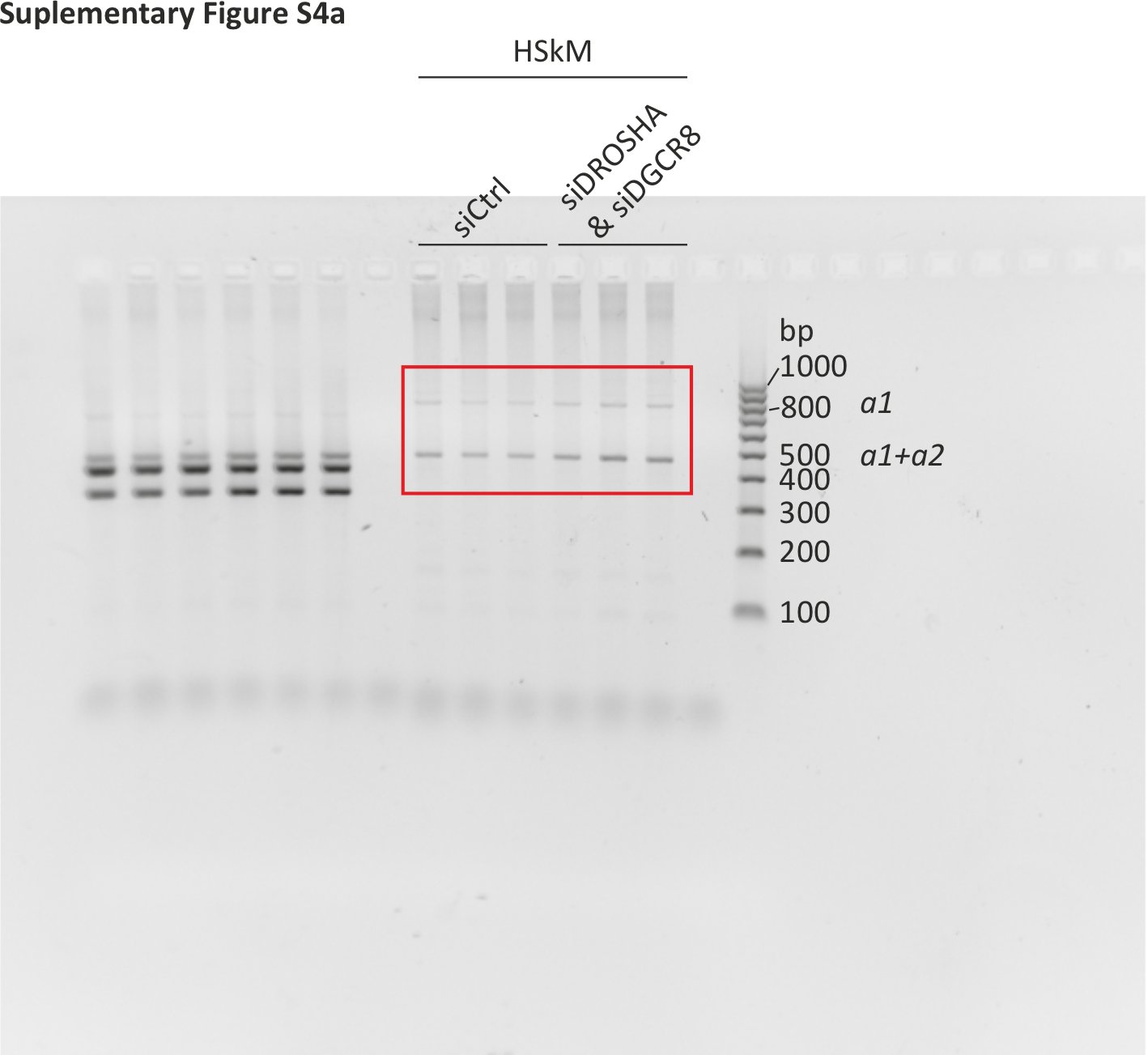


**Fig. SXV. Full size image of the blot corresponding to Supplementary Fig. S4a.**

Gel, *CELF1* 3’UTR isoforms amplification after DROSHA and DGCR8 knock-down. Red rectangle, bands used to make the manuscript figures.


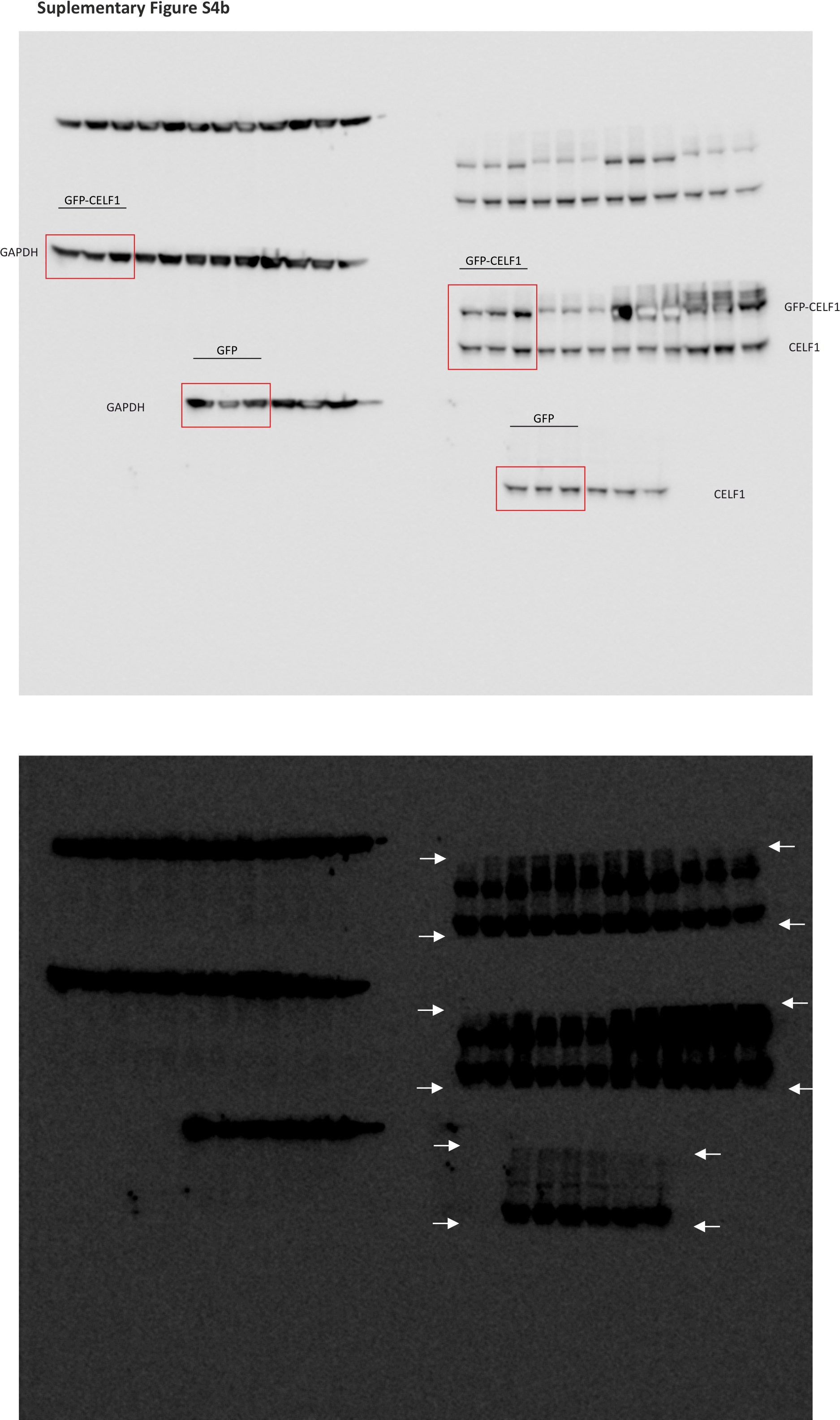


**Fig. SXVI. Full size images of the blot corresponding to Supplementary Fig. S4b.**

Western blots with anti-CELF1 antibody and anti-GAPDH as loading control. Original images (upper panel) and high contrasted images (lower panel) are presented; membrane edges are indicated with arrows; some edges are not visible due to short exposure time. Red rectangle, bands used to make the manuscript figures.


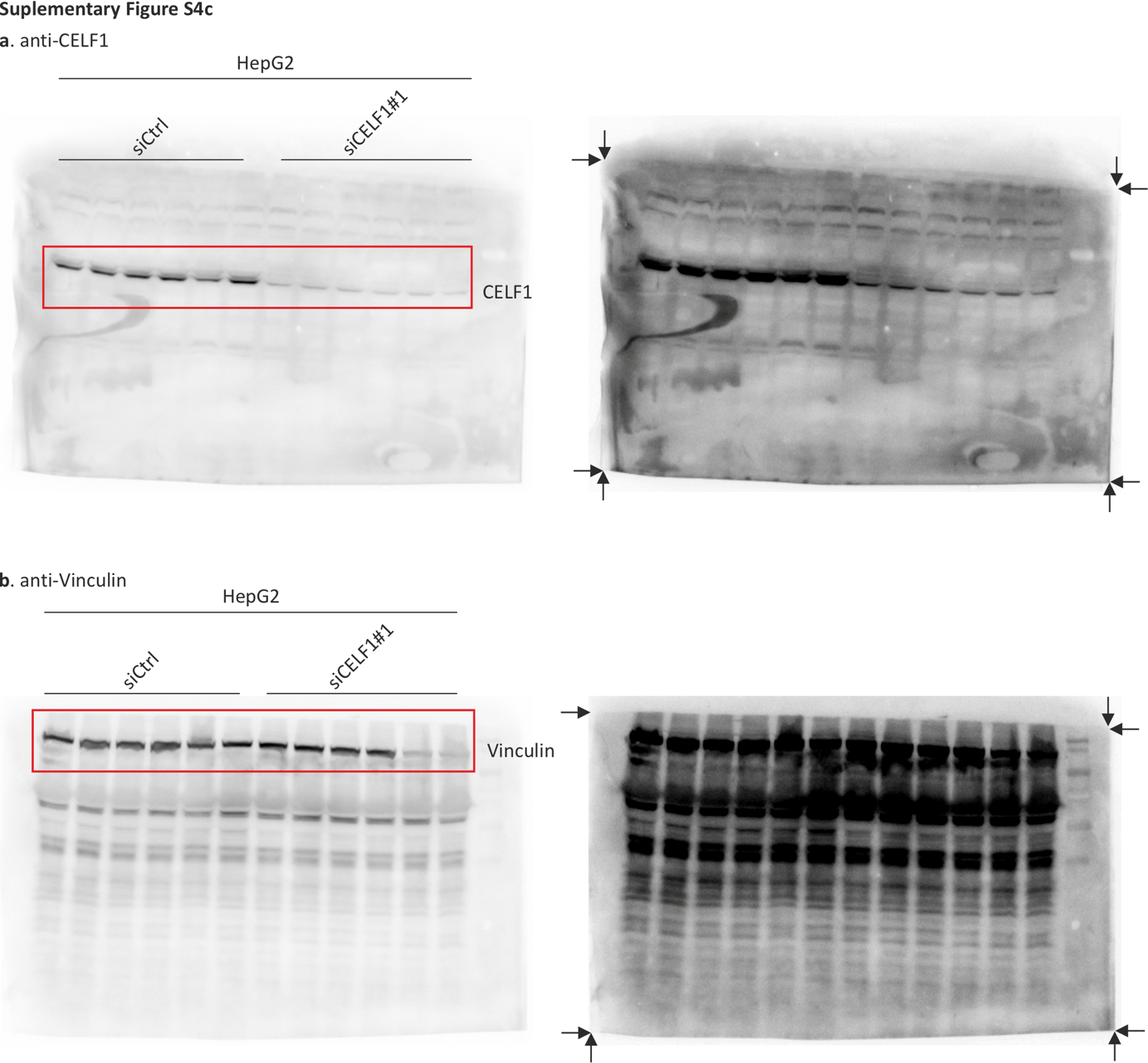


**Fig. SXVII. Full size images of the blot corresponding to Supplementary Fig. S4c.**

Western blots, CELF1 (a) and Vinculin (b) as loading control after *CELF1* knock-down with siRNA set #1 in HepG2 cells. Original images (left panel) and high contrasted images (right panel) are presented; membrane edges are indicated with arrows. Red rectangle, bands used to make the manuscript figures.


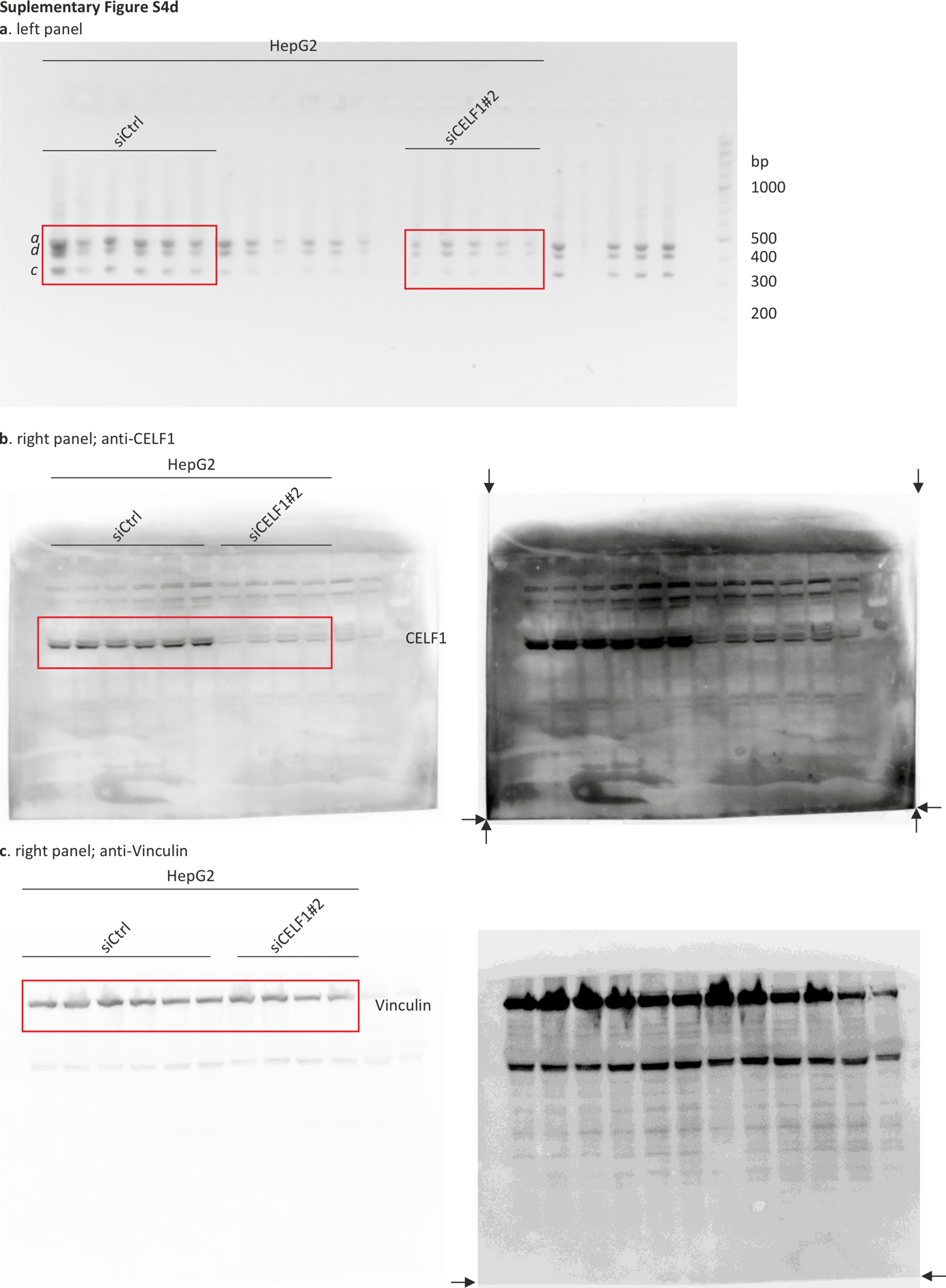


**Fig. SXVIII. Full size images of the blot corresponding to Supplementary Fig. S4d.**

(a) Gels, *CELF1* 3’UTR isoforms amplification after *CELF1* knock-down with siRNA set #2 in HepG2 cells.

(b, c) Western blots, CELF1 (b) and Vinculin (c) as loading control after *CELF1* knock-down with siRNA set #2 in HepG2 cells. Original images (left panel) and high contrasted images (right panel) are presented; membrane edges are indicated with arrows.

Red rectangle, bands used to make the manuscript figures.

**Supplementary Table S1.** *CELF1* ex5 inclusion level in RNA-Seq data sets after MBNL1 or MBNL2 knock down with specific siRNA or shRNA. Inclusion level difference, p-value and FDR are calculated with MATS (v4.1). Bold, *p*-value and FDR lower than 0.05.

| **GEO accession number** | **Treatment** | ***CELF1* ex5 inclusion level difference (MATS)** | ***CELF1* ex5 inclusion *p‑*value (MATS)** | ***CELF1* ex5 inclusion FDR (MATS)** |
| --- | --- | --- | --- | --- |
| GSE149435 | siMBNL1 in MDA-MB-231 cell line | -0.247 | **0.001241244** | 0.050365682 |
| GSE136231 | siMBNL2 in MCF-7 cell line | -0.241 | **5.78E-05** | **0.002733009** |
| GSE123441 | shMBNL1 in MOLM-13 cell line | -0.221 | **0.000724951** | **0.029758135** |
| GSE123441 | shMBNL1#65 in MOLM-13 cell line | -0.095 | 0.238204012 | 1 |
| GSE123441 | shMBNL1#64 in MV4 cell line | -0.326 | **1.32E-10** | **3.93E-08** |
| GSE123441 | shMBNL1#65 in MV4 cell line | -0.288 | **5.84E-08** | **2.36E-05** |
| GSE83999 | shMBNL2 in 786-O cell line | 0.038 | 0.341399753 | 1 |
| GSE83999 | shMBNL2 in A-498 cell line | -0.216 | **4.43E-05** | **0.003283151** |

**Supplementary Table S2.** Primers, siRNAs and oligonucleotides used in the experimental procedures. The localization of primers is presented in Supplementary Fig. S1.

| **Name** | **Sequence 5’→3’** | **RS** | **Tm**  **(^o^C)** | **Description** |
| --- | --- | --- | --- | --- |
| **siRNA oligonucleotides and 5MCS-pmirGLO oligonucleotides** | | | | |
| siCELF1#1 | p-GUUACGACAAUCCUGUUUCdTdT |  |  | siCELF1 sense #1 |
| siCELF1#1 | p-GAAACAGGAUUGUCGUAACdTdT |  |  | siCELF1 antisense #1 |
| siCELF1#2 | p-GAGCCAACCUGUUCAUCUAdTdT |  |  | siCELF1 sense #2 |
| siCELF1#2 | p-UAGAUGAACAGGUUGGCUCdTdT |  |  | siCELF1 antisense #2 |
| siMBNL1#1 | p-CACUGGAAGUAUGUAGAGAdTdT |  |  | siMBNL1 sense #1 |
| siMBNL1#1 | p-UCUCUACAUACUUCCAGUGdTdT |  |  | siMBNL1 antisense #1 |
| siMBNL2#1 | p-GAGGAACAUGCUCACGCUCdTdT |  |  | siMBNL2 sense #1 |
| siMBNL2#1 | p-GAGCGUGAGCAUGUUCCUCdTdT |  |  | siMBNL2 antisense #1 |
| siMBNL1#2 | p-GGACGAGGUCAUUAGCCAUdTdT |  |  | siMBNL1 sense #2 |
| siMBNL1#2 | p-AUGGCUAAUGACCUCGUCCdTdT |  |  | siMBNL1 antisense #2 |
| siMBNL2#2 | p-CACCGUAACCGUUUGUAUGdTdT |  |  | siMBNL2 sense #2 |
| siMBNL2#2 | p-CAUACAAACGGUUACGGUGdTdT |  |  | siMBNL2 antisense #2 |
| siDROSHA | p-CGAGUAGGCUUCGUGACUUdTdT |  |  | siDROSHA sense |
| siDROSHA | p-AAGUCACGAAGCCUACUCGdUdU |  |  | siDROSHA antisense |
| siDGCR8 | p-CAUCGGACAAGAGUGUGAUdTdT |  |  | siDGCR8 sense |
| siDGCR8 | p-AUCACACUCUUGUCCGAUGdUdU |  |  | siDGCR8 antisense |
| siCtrl | p-UAAGGCUAUGAAGAGAUACdTdT |  |  | siCtrl sense |
| siCtrl | p-GUAUCUCUUCAUAGCCUUAdTdT |  |  | siCtrl antisense |
| 5MCS-pmirGLO | AGCTTCATATGCCCGGGGAATTCA |  |  | 5MCS-pmirGLO sense oligo |
| 5MCS-pmirGLO | AGCTTGAATTCCCCGGGCATATGA |  |  | 5MCS-pmirGLO antisense oligo |
| **Cloning and minigenes primers** | | | | |
|  | TGCACGCTCGAGCCATGAACGGCACCCTGGACCA | *Xho*I | 55 | Forward primer for amplification of the shorter *Celf1* isoform |
|  | TGCACGCTCGAGCCATGGCTTACCCATACGAT | *Xho*I | 55 | Forward primer for amplification of the longer *Celf1* isoform |
|  | TGCACGGGATCCTCAGAGGGGAGCACTCAGTA | *BamH*I | 55 | Reverse primer for amplification of both *Celf1* isoforms |
|  | TGCACGCATATGGGATTCGGCCTCAGCAGCGA | *Nde*I | 55 | Forward primer for amplification of all *CELF1* 5’UTR isoforms |
|  | TGCACGGAATTCGGGTGCCGTTCACTTTCTTTGAG | *EcoR*I | 55 | Reverse primer for amplification of the *CELF1* AUG2 5’UTR isoform |
|  | TGCACGGAATTCCAACTTAAACGCAGCCACCACC | *EcoR*I | 55 | Reverse primer for amplification of the *CELF1* AUG1 5’UTR isoform |
|  | TGCACGCATATGGAAGCTAAAGCTGTGTGTATTTG | *Nde*I | 55 | Forward primer for amplification of the *CELF1* AUG1 5’UTR isoform – codon start context |
|  | TGCACGGAATTCTAAACGCAGCCATCACCTCAC | *EcoR*I | 55 | Reverse primer for amplification of the *CELF1* AUG1 5’UTR isoform – codon start context |
|  | TGCACGCATATGCGAGGCGGCGGCGGCGGCTG | *Nde*I | 55 | Forward primer for amplification of the *CELF1* AUG2 5’UTR isoform – codon start context |
|  | TGCACGGAATTCGGGTGCCGTTCATTTTCTTTGAG | *EcoR*I | 55 | Reverse primer for amplification of the *CELF1* AUG2 5’UTR isoform– codon start context |
|  | TGCACGGCTAGCTCCCCTCTGAGACTGGAGTG | *Nhe*I | 55 | Forward primer for amplification of *CELF1* 3’UTR isoforms *a1* and *d* |
|  | TGCACGCTCGAGAAGCACACAGAGAAACAAAGC | *Xho*I | 55 | Reverse primer for amplification of *CELF1* 3’UTR isoform *a1* |
|  | TGCACGTCTAGAGGACACATTTCTCCCAGTGC | *Xba*I | 55 | Reverse primer for amplification of *CELF1* 3’UTR isoform *d* |
|  | TGCACGTTTAAACTGCCGTTTGTTCATCGTT | *Pme*I | 55 | Forward primer for amplification of *CELF1* 3’UTR isoform *a2* – fragment 1 |
|  | TGCACGGCTAGCCTGCTTCTCGTGTCTCTCC | *Nhe*I | 55 | Reverse primer for amplification of *CELF1* 3’UTR isoform *a2* – fragment 1 |
|  | TGCACGGCTAGCAGGTAGAGCCTAGAAGGTG | *Nhe*I | 55 | Forward primer for amplification of *CELF1* 3’UTR isoform *a2* – fragment 2 |
|  | TGCACGCTCGAGTTGTCCATGTGTCTGTGCA | *Xho*I | 55 | Reverse primer for amplification of *CELF1* 3’UTR isoform *a2* – fragment 2 |
| pcDNA_CELF1_F | CTAGAACAAAAACTCATCTCAGAAG |  | 55 |  |
| pcDNA_CELF1_R | AGCTTGGGTCTCCCTATAGTG |  | 55 |  |
| CELF1_AUG1_F | AGGGAGACCCAAGCTATGGCTGCGTTTAAGTTGG |  | 55 |  |
| CELF1_R | GAGTTTTTGTTCTAGGTAGGGCTTACTATCATTCTTCG |  | 55 |  |
| CELF1_AUG2_F | AGGGAGACCCAAGCTATGAACGGCACCCTGGAC |  | 55 |  |
| pcDNA_IRES_F | CAGCCTCGACTGTGCCTTC |  | 55 |  |
| pcDNA_IRES_R | AACTCAATGATGATGATGATGATGG |  | 55 |  |
| IRES_F | CATCATCATTGAGTTACGTTACTGGCCGAAGCCG |  | 55 |  |
| IRES_R | GCACAGTCGAGGCTGTTACTTGTACAGCTCGTCCATGCC |  | 55 |  |
| NF1_F | TTGACCATTCACCACATTGGTGTGC |  | 55 | *NF1* minigene |
| NF1_R | ATCTGGGAGCGGGGCTGT |  | 55 | *NF1* minigene |
| cTNT_F | CATTCACCACATTGGTGTGC |  | 55 | *cTNT* minigene |
| cTNT_R | AGGTGCTGCCGCCGGGCGGTGGCTG |  | 55 | *cTNT* minigene |
| ***CELF1* 5’UTR RT-PCR analysis primers** | | | | |
| 5_F1 | GGATTCGGCCTCAGCAGCGA |  | 60 |  |
| 5_F2 | TGTGGAGGACTGAGGTTTTCA |  | 60 |  |
| 5_F3 | TGATGGCTGCGTTTAAGTTG |  | 60 |  |
| 5_F4 | ATGAACGGCACCCTGGAC |  | 60 |  |
| 5_R | TGCTCTGAGGCGGGTTTTGG |  | 60 |  |
| 5_R1 | AATGCAGCTTTACGGGTGTA |  | 60 |  |
| ***CELF1* 3’UTR RT-PCR analysis primers** | | | | |
| 3_F1 | GCCCTTTGGGAATGTCGTGTC |  | 55 |  |
| 3_Rcd | CTCCAAAGTAAAACACTGGAAACC |  | 55 |  |
| 3_Ra | AGCAGTCGTTCTAGCAAAACCA |  | 55 |  |
| 3_Rb | GGACACATTTCTCCCAGTGC |  | 55 |  |
| 3_R2a | ACATGCGGTCTCCTCAAGC |  | 55 |  |
| 3_F2 | TGGATTATCCCATTGAAGCTG |  | 55 |  |
| 3_R2b | ATCCCATCATGACCCTGGA |  | 55 |  |
| **Alternative splicing CELF-dependent exon primers** | | | | |
| CAPZB_F | GTACGCTGAACGAGATCTACTTTG |  | 55 | Localized in exon 7 |
| CAPZB_R | CTGGTTAGCATGAAACAGAG |  | 55 | Localized in exon 9 |
| ANK2_F | AGAGCACAGAGAGGAGAGCT |  | 55 | Localized in exon 20 |
| ANK2_R | TCTTCTGTGACTGTTTCTGG |  | 55 | Localized in exon 22 |
| ITGB1_F | CCCACTGGTCCAGACATCATT |  | 55 | Localized in exon 16 |
| ITGB1_R | CTTCGGATTGACCACAGTTGTT |  | 55 | Localized in exon 18 |
| **mRNA expression level analysis primers** | | | | |
| CELF1_F | CCAAGGACCTGGTCTGAAAA |  | 60 |  |
| CELF1_R | ATCCCTGGGAGGACTTTCAT |  | 60 |  |
| ACTB_F | ACAGAGCCTCGCCTTTGCCGAT |  | 60 |  |
| ACTB_R | ATCATCCATGGTGAGCTGGCGG |  | 60 |  |
| GFP_F | AGGACGACGGCAACTACAAG |  | 60 |  |
| GFP_R | TTCTGCTTGTCGGCCATGAT |  | 60 |  |
| GAPDH_F | CATCAATGGAAATCCCATCAC |  | 60 |  |
| GAPDH_R | GGTTTTTCTAGACGGCAGGTC |  | 60 |  |

F, forward primer; R, reverse primer; P, transcription promoter; APAS, alternative polyadenylation site; underline, restriction site; RS, restriction site; Tm, melting temperature

**Supplementary Table S3.** Antibodies used in western blots.

| **Name** | **Clone** | **Producer (cat. no)** | **Dilution** | **Secondary antibody** | **Producer (cat. no)** | **Dilution** |
| --- | --- | --- | --- | --- | --- | --- |
| Anti-CELF1 | 3B1 | Millipore (05-621) | 1:1000 | goat anti-mouse | Millipore (12-349) | 1:10000 |
| Anti-MBNL1 (Holt *et al*, 2009) | 4A8 |  | 1:1000 | goat anti-mouse | Millipore (12-349) | 1:10000 |
| Anti-MBNL2 (Holt *et al*, 2009) | 3B4 |  | 1:500 | goat anti-mouse | Millipore (12-349) | 1:40000 |
| Anti-Myc/c-Myc | 9E10 | Santa Cruz Biotechnology (sc-40) | 1:1000 | goat anti-mouse | Millipore (12-349) | 1:10000 |
| Anti-GAPDH | 0411 | Santa Cruz Biotechnology (sc-47724) | 1:10000 | goat anti-mouse | Millipore (12-349) | 1:20000 |
| Anti-VINCULIN |  | CST (4650) | 1:1000 | goat anti-rabbit | Sigma (A9169) | 1:10000 |

**Supplementary Table S4.** *P*-values and statistical test for all comparisons presented in current work.

| **Figure** | **Samples** | ***p*-value** | **Statistical test** |
| --- | --- | --- | --- |
| Fig. 1d | Skeletal muscles ex4, non-DM vs DM1 (50-75) | 0.002118766 | Unpaired, two-tailed *t*-test |
| Fig. 1d | Skeletal muscles ex4, non-DM vs DM1 (25-50) | 0.016856031 | Unpaired, two-tailed *t*-test |
| Fig. 1d | Skeletal muscles ex4, non-DM vs DM1 (0-25) | 0.016856031 | Unpaired, two-tailed *t*-test |
| Fig. 1d | Skeletal muscles ex4, non-DM vs DM1 | 7.05411E-06 | Unpaired, two-tailed *t*-test |
| Fig. 1d | Skeletal muscles ex5, non-DM vs DM1 (0-25) | 0.001640025 | Unpaired, two-tailed *t*-test |
| Fig. 1d | Skeletal muscles ex5, non-DM vs DM1 | 0.039376877 | Unpaired, two-tailed *t*-test |
| Fig. 1d | Heart ex5, non-DM vs DM1 | 0.027706382 | Unpaired, two-tailed *t*-test |
| Fig. 1g | *CELF1* ex5, siCtrl vs siMBNL1&2#1 | 0.0037 | Unpaired, two-tailed *t*-test |
| Fig. 1g | *CELF1* % of isoform *a* , siCtrl vs siMBNL1&2#1 | 0.023117 | Unpaired, two-tailed *t*-test |
| Fig. 1g | CELF1 protein, siCtrl vs siMBNL1&2#1 | 0.02066701 | Unpaired, two-tailed *t*-test |
| Fig. 2a | Muscles vs Heart | 1.726E-155 | Unpaired, two-tailed *t*-test |
| Fig. 2a | Muscles vs Brain | 0 | Unpaired, two-tailed *t*-test |
| Fig. 2a | Brain vs Heart | 4.0631E-105 | Unpaired, two-tailed *t*-test |
| Fig. 2d | Skeletal muscles (1 day) vs Skeletal muscles (90 days) | 0.00940426 | Unpaired, two-tailed *t*-test |
| Fig. 2d | Heart (1 day) vs Heart (90 days) | 0.18658323 | Unpaired, two-tailed *t*-test |
| Fig. 2d | Brain (1 day) vs Brain (90 days) | 8.7114E-08 | Unpaired, two-tailed *t*-test |
| Fig. 2e | Skeletal muscles (1 day) vs Skeletal muscles (90 days), isoforms *c*+*d* | 0.048214 | Unpaired, two-tailed *t*-test |
| Fig. 2e | Skeletal muscles (1 day) vs Skeletal muscles (90 days), isoforms *a1*+*a2* | 0.001513 | Unpaired, two-tailed *t*-test |
| Fig. 2e | Skeletal muscles (1 day) vs Skeletal muscles (90 days), isoforms *a2* | 0.000616 | Unpaired, two-tailed *t*-test |
| Fig. 2e | Heart (1 day) vs Heart (5 days), isoforms *a1*+*a2* | 0.005166 | Unpaired, two-tailed *t*-test |
| Fig. 2e | Heart (1 day) vs Heart (14 days), isoforms *a1*+*a2* | 0.006794 | Unpaired, two-tailed *t*-test |
| Fig. 2e | Heart (1 day) vs Heart (90 days), isoforms *a1*+*a2* | 0.00474 | Unpaired, two-tailed *t*-test |
| Fig. 2e | Heart (1 day) vs Heart (5 days), isoforms *a2* | 0.005815 | Unpaired, two-tailed *t*-test |
| Fig. 2e | Heart (1 day) vs Heart (14 days), isoforms *a2* | 0.000154 | Unpaired, two-tailed *t*-test |
| Fig. 2e | Heart (1 day) vs Heart (90 days), isoforms *a2* | 0.000436 | Unpaired, two-tailed *t*-test |
| Fig. 2e | Brain (1 day) vs Brain (5 days), isoforms *a1*+*a2* | 1.94E-06 | Unpaired, two-tailed *t*-test |
| Fig. 2e | Brain (1 day) vs Brain (14 days), isoforms *a1*+*a2* | 3.65E-07 | Unpaired, two-tailed *t*-test |
| Fig. 2e | Brain (1 day) vs Brain (90 days), isoforms *a1*+*a2* | 7.9E-08 | Unpaired, two-tailed *t*-test |
| Fig. 2e | Brain (1 day) vs Brain (5 days), isoforms *a2* | 0.00038622 | Unpaired, two-tailed *t*-test |
| Fig. 2e | Brain (1 day) vs Brain (14 days), isoforms *a2* | 4.96889E-05 | Unpaired, two-tailed *t*-test |
| Fig. 2e | Brain (1 day) vs Brain (90 days), isoforms *a2* | 1.03795E-05 | Unpaired, two-tailed *t*-test |
| Fig. 3a | i1-Luc vs i5-Luc | 0.0205 | Unpaired, two-tailed *t*-test |
| Fig. 3f | *NF1* CELF1-AUG1 30ng vs CELF1-AUG2 30ng | 0.0097 | Unpaired, two-tailed *t*-test |
| Fig. 3f | *NF1* CELF1-AUG1 60ng vs CELF1-AUG2 60ng | 0.0244 | Unpaired, two-tailed *t*-test |
| Fig. 3f | *NF1* CELF1-AUG1 125ng vs CELF1-AUG2 125ng | 0.0062 | Unpaired, two-tailed *t*-test |
| Fig. 3f | *cTNT* CELF1-AUG1 30ng vs CELF1-AUG2 30ng | 0.2734 | Unpaired, two-tailed *t*-test |
| Fig. 3f | *cTNT* CELF1-AUG1 60ng vs CELF1-AUG2 60ng | 0.0037 | Unpaired, two-tailed *t*-test |
| Fig. 3f | *cTNT* CELF1-AUG1 125ng vs CELF1-AUG2 125ng | 0.0164 | Unpaired, two-tailed *t*-test |
| Fig. 4a | HeLa 3’UTR-*a1* vs 3’UTR-*a2* | 4.03363E-05 | Unpaired, two-tailed *t*-test |
| Fig. 4a | HeLa 3’UTR-*d* vs 3’UTR-*a2* | 0.000848347 | Unpaired, two-tailed *t*-test |
| Fig. 4a | HeLa 3’UTR-*a1* vs 3’UTR-*d* | 1.23231E-05 | Unpaired, two-tailed *t*-test |
| Fig. 4a | HepG2 3’UTR-*a1* vs 3’UTR-*a2* | 0.003334 | Unpaired, two-tailed *t*-test |
| Fig. 4a | HepG2 3’UTR-*d* vs 3’UTR-*a2* | 0.004217 | Unpaired, two-tailed *t*-test |
| Fig. 4a | HepG2 3’UTR-*a1* vs 3’UTR-*d* | 0.000951 | Unpaired, two-tailed *t*-test |
| Fig. 4a | COS-7 3’UTR-*a1* vs 3’UTR-*a2* | 4.42645E-06 | Unpaired, two-tailed *t*-test |
| Fig. 4a | COS-73’UTR-*d* vs 3’UTR-*a2* | 0.007198202 | Unpaired, two-tailed *t*-test |
| Fig. 4a | COS-73’UTR-*a1* vs 3’UTR-*d* | 0.000239842 | Unpaired, two-tailed *t*-test |
| Fig. 4b | siCtrl vs siDROSHA & siDGCR8 | 0.020293 | Unpaired, two-tailed *t*-test |
| Fig. 4c | GFP vs CELF1 | 0.0014 | Unpaired, two-tailed *t*-test |
| Fig. 4c | siCtrl vs siCELF1#1 | 0.0220 | Unpaired, two-tailed *t*-test |
| Fig. 4d | WT vs 24h | 0.0115 | Unpaired, two-tailed *t*-test |
| Fig. 4d | WT vs 72h | 0.0054 | Unpaired, two-tailed *t*-test |
| Fig. 4d | WT vs 7d | 0.0021 | Unpaired, two-tailed *t*-test |
| Fig. 4f | Muscle vs Heart | 1.43419E-61 | Unpaired, two-tailed *t*-test |
| Fig. 4f | Muscle vs Brain | 0.003079852 | Unpaired, two-tailed *t*-test |
| Fig. 4f | Brain vs Heart | 4.10051E-59 | Unpaired, two-tailed *t*-test |
| Fig. 4g | *ANK2* ex21 Muscle vs Heart | 7.4196E-24 | Unpaired, two-tailed *t*-test |
| Fig. 4g | *ANK2* ex21 Muscle vs Brain | 0 | Unpaired, two-tailed *t*-test |
| Fig. 4g | *ANK2* ex21 Brain vs Heart | 0 | Unpaired, two-tailed *t*-test |
| Fig. 4g | *CAPZB* ex8 Muscle vs Heart | 2.1808E-229 | Unpaired, two-tailed *t*-test |
| Fig. 4g | *CAPZB* ex8 Muscle vs Brain | 0 | Unpaired, two-tailed *t*-test |
| Fig. 4g | *CAPZB* ex8 Brain vs Heart | 6.2974E-242 | Unpaired, two-tailed *t*-test |
| Fig. 4g | *ITGB1* ex17 Muscle vs Heart | 9.047E-145 | Unpaired, two-tailed *t*-test |
| Fig. 4g | *ITGB1* ex17 Muscle vs Brain | 0 | Unpaired, two-tailed *t*-test |
| Fig. 4g | *ITGB1* ex17 Brain vs Heart | 0 | Unpaired, two-tailed *t*-test |
| Fig. 4g | *MTMR3* ex16 Muscle vs Heart | 2.566E-216 | Unpaired, two-tailed *t*-test |
| Fig. 4g | *MTMR3* ex16 Muscle vs Brain | 0 | Unpaired, two-tailed *t*-test |
| Fig. 4g | *MTMR3* ex16 Brain vs Heart | 0 | Unpaired, two-tailed *t*-test |
| Fig. 4g | *RBFOX2* ex13 Muscle vs Heart | 7.6377E-93 | Unpaired, two-tailed *t*-test |
| Fig. 4g | *RBFOX2* ex13 Muscle vs Brain | 0 | Unpaired, two-tailed *t*-test |
| Fig. 4g | *RBFOX2* ex13 Brain vs Heart | 0 | Unpaired, two-tailed *t*-test |
| Fig. 4g | *MINDY3* ex5 Muscle vs Heart | 2.451E-218 | Unpaired, two-tailed *t*-test |
| Fig. 4g | *MINDY3* ex5 Muscle vs Brain | 0 | Unpaired, two-tailed *t*-test |
| Fig. 4g | *MINDY3* ex5 Brain vs Heart | 2.168E-307 | Unpaired, two-tailed *t*-test |
| Supplementary Fig. S2a | GFP vs MBNL1 | 0.0163112 | Unpaired, two-tailed *t*-test |
| Supplementary Fig. S2a | siCtrl vs MBNL1&2#1 | 0.041802 | Unpaired, two-tailed *t*-test |
| Supplementary Fig. S2b | GFP vs MBNL1 | 0.000917 | Unpaired, two-tailed *t*-test |
| Supplementary Fig. S2c | HSkM, MBNL1, siCtrl vs MBNL1&2#1 | 0.01730968 | Unpaired, two-tailed *t*-test |
| Supplementary Fig. S2c | HSkM, MBNL2, siCtrl vs MBNL1&2#1 | 0.03478087 | Unpaired, two-tailed *t*-test |
| Supplementary Fig. S2c | HeLa, MBNL1, siCtrl vs MBNL1&2#1 | 0.00158 | Unpaired, two-tailed *t*-test |
| Supplementary Fig. S2c | HeLa, MBNL2, siCtrl vs MBNL1&2#1 | 0.000577 | Unpaired, two-tailed *t*-test |
| Supplementary Fig. S2d | *CELF1* ex5, siCtrl vs MBNL1&2#2 | 0.0163 | Unpaired, two-tailed *t*-test |
| Supplementary Fig. S2d | *CELF1* % of isoform *a*, siCtrl vs MBNL1&2#2 | 0.0300 | Unpaired, two-tailed *t*-test |
| Supplementary Fig. S2d | CELF1 protein, siCtrl vs MBNL1&2#2 | 0.0280 | Unpaired, two-tailed *t*-test |
| Supplementary Fig. S2e | HepG2, MBNL1, siCtrl vs MBNL1&2#2 | 0.0001 | Unpaired, two-tailed *t*-test |
| Supplementary Fig. S2e | HepG2, MBNL2, siCtrl vs MBNL1&2#2 | <0.0001 | Unpaired, two-tailed *t*-test |
| Supplementary Fig. S3a | GFP Luc vs CELF1 Luc | 0.0014 | Unpaired, two-tailed *t*-test |
| Supplementary Fig. S4a | siCtrl vs siDROSHA & siDGCR8 | 0.0203 | Unpaired, two-tailed *t*-test |
| Supplementary Fig. S4c | HepG2, CELF1, siCtrl vs CELF1#1 | <0.0001 | Unpaired, two-tailed *t*-test |
| Supplementary Fig. S4d | *CELF1* % of isoform *a*, siCtrl vs CELF1#2 | 0.0113 | Unpaired, two-tailed *t*-test |
| Supplementary Fig. S4d | HepG2, CELF1, siCtrl vs CELF1#2 | 0.0001 | Unpaired, two-tailed *t*-test |

**Supplementary references**

1 Sznajder, Ł. *et al.* Mechanistic determinants of MBNL activity. *Nucleic Acids Res* **44**, 10326-10342, doi:10.1093/nar/gkw915 (2016).
